# Subcellular mRNA Localization Regulates Ribosome Biogenesis in Migrating Cells

**DOI:** 10.1016/j.devcel.2020.10.006

**Published:** 2020-11-09

**Authors:** Maria Dermit, Martin Dodel, Flora C.Y. Lee, Muhammad S. Azman, Hagen Schwenzer, J. Louise Jones, Sarah P. Blagden, Jernej Ule, Faraz K. Mardakheh

**Affiliations:** 1 Centre for Cancer Cell and Molecular Biology, Barts Cancer Institute, Queen Mary University of London, Charterhouse Square, London EC1M 6BQ, UK; 2 The Francis Crick Institute, 1 Midland Road, London NW1 1AT, UK; 3 Department of Neuromuscular Diseases, UCL Queen Square Institute of Neurology, Queen Square, London WC1N 3BG, UK; 4 Department of Oncology, University of Oxford, Oxford OX3 7DQ, UK; 5 Centre for Tumour Biology, Barts Cancer Institute, Queen Mary University of London, Charterhouse Square, London EC1M 6BQ, UK

**Keywords:** RNA localization, ribosome biogenesis, La-related proteins, EMT, invasion, cancer, LARP6, ribosomal proteins, protrusion

## Abstract

Translation of ribosomal protein-coding mRNAs (RP-mRNAs) constitutes a key step in ribosome biogenesis, but the mechanisms that modulate RP-mRNA translation in coordination with other cellular processes are poorly defined. Here, we show that subcellular localization of RP-mRNAs acts as a key regulator of their translation during cell migration. As cells migrate into their surroundings, RP-mRNAs localize to the actin-rich cell protrusions. This localization is mediated by La-related protein 6 (LARP6), an RNA-binding protein that is enriched in protrusions. Protrusions act as hotspots of translation for RP-mRNAs, enhancing RP synthesis, ribosome biogenesis, and the overall protein synthesis in migratory cells. In human breast carcinomas, epithelial-to-mesenchymal transition (EMT) upregulates LARP6 expression to enhance protein synthesis and support invasive growth. Our findings reveal LARP6-mediated mRNA localization as a key regulator of ribosome biogenesis during cell migration and demonstrate a role for this process in cancer progression downstream of EMT.

## Highlights

• Translation of RP-mRNAs is a key step in ribosome biogenesis
• In migrating cells, LARP6 localizes RP-mRNAs to actin-rich cell protrusions
• Protrusions act as hotspots of RP-mRNA translation, enhancing ribosome biogenesis
• LARP6 expression is associated with EMT and upregulated in aggressive carcinomas

## Graphical Abstract

**Figure:**
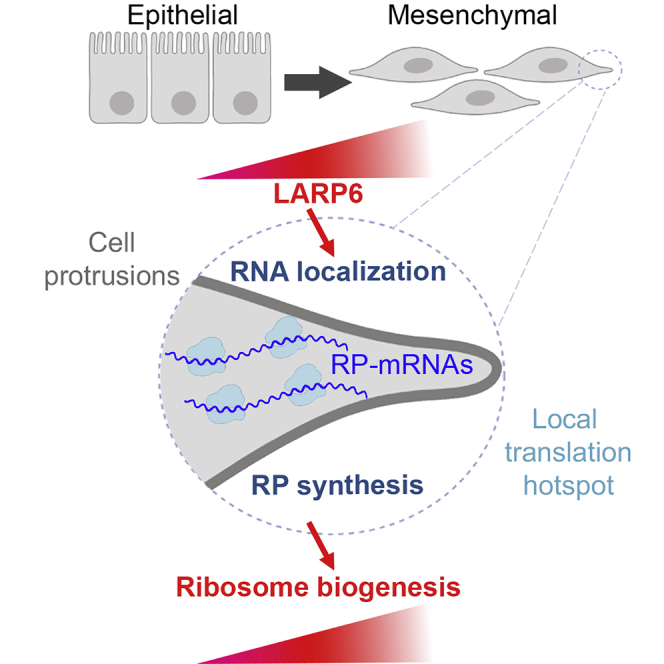

## Teaser

Dermit et al. reveal that ribosomal protein (RP)-mRNAs localize to the protrusive fronts of migratory cells, where their translation is locally increased, leading to upregulation of ribosome biogenesis and protein synthesis. In aggressive carcinomas, this pathway is upregulated in order to support the high anabolic demands of invasive cancer cells.

## Introduction

Ribosome biogenesis, the highly conserved process of synthesis, processing, and assembly of ribosomal RNA (rRNA) and protein (RP) components into mature ribosomes (Bohnsack and Bohnsack, 2019), underpins all protein synthesis in living organisms. In parallel with RNA-polymerase-I-dependent regulation of rRNA transcription, translation of RP-coding mRNAs (RP-mRNAs) acts as a key step in control of ribosome biogenesis in higher eukaryotes (Gentilella et al., 2015). Mechanistic target of rapamycin complex 1 (mTORC1) has been shown to regulate RP-mRNA synthesis downstream of growth factor stimulation or nutrient availability, through phosphorylating and modulating the interaction of an evolutionary conserved RNA-binding protein (RBP) named La-related protein-1 (LARP1) with RP-mRNAs (Fonseca et al., 2015; Tcherkezian et al., 2014). LARP1 directly interacts with RP-mRNAs via multiple sites, including the 5′ terminal oligo pyrimidine (TOP) motif, a stretch of 6–12 pyrimidines present at the 5′ end of transcripts that code for components of the translation machinery, as well as the 5′ mRNA cap, the 3′ untranslated region (3′UTR), and the Poly-A tail (Al-Ashtal et al., 2019; Hong et al., 2017; Lahr et al., 2017). A recent model proposes that mTORC1 phosphorylation acts as a molecular switch, converting LARP1 from a translational inhibitor to activator, leading to upregulation of RP-mRNAs translation and subsequent ribosome biogenesis (Hong et al., 2017). Nevertheless, it is unclear whether other cellular processes can regulate RP-mRNA translation, independently of the mTORC1-LARP1 pathway, in response to further intrinsic or extrinsic inputs.

Mesenchymal-like cell migration is a highly resource intensive cellular process that requires production of large quantities of actin cytoskeletal, cell adhesion, and extracellular matrix proteins, many of which are among the most abundant proteins in the proteome of mammalian cells (Schwanhäusser et al., 2011). Polarization of cells into a protrusive front and a retractile back is the defining feature of mesenchymal-like migration. Interestingly, a number of studies have reported that RP-mRNAs can strongly localize to the protrusive fronts of some mesenchymal-like cells (Mardakheh et al., 2015; Mili et al., 2008; Wang et al., 2017). Nevertheless, the molecular mechanism as well as the functional significance of this localization has remained unclear.

Here, we employed a subcellular multi-omics analysis to demonstrate that RP-mRNA localization to protrusive fronts is a universal feature of mesenchymal-like migrating cells. This localization is mediated via LARP6, a microtubule-associated homolog of LARP1 that directly binds to RP-mRNAs to promote their enrichment in protrusions, independent of mTORC1 activity. Protrusive fronts are also highly enriched in translation initiation and elongation factors, acting as hotspots for translation of localized RP-mRNAs. LARP6-dependent localization of RP-mRNAs results in upregulation of RP synthesis, leading to enhancement of ribosome biogenesis and increased protein synthetic capacity required to support sustained migration and proliferation of highly motile cells. In human breast carcinomas, higher LARP6 expression is associated with the invasive mesenchymal-like subtypes. Epithelial-to-mesenchymal transition (EMT) induces LARP6 expression, which acts to promote protein synthesis in order to enhance malignant cell proliferation and invasion. Our findings reveal a mechanism that governs ribosome biogenesis in mesenchymal-like migratory cells via subcellular localization of RP-mRNAs, and demonstrate a targetable role for this process in aggressive cancers downstream of EMT.

## Results

### RP-mRNAs Localize to Protrusions of All Migratory Cells

**Figure 1:**
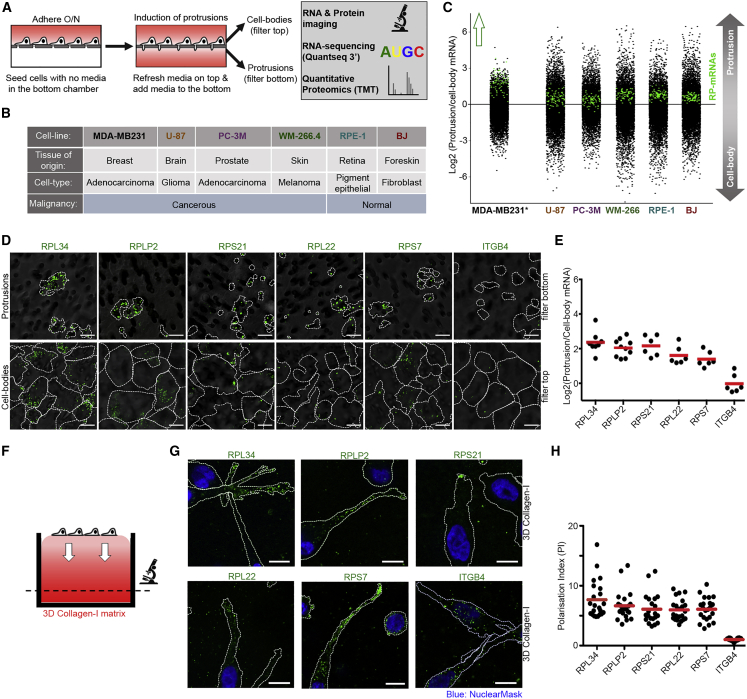
RP-mRNAs Localize to Protrusions of All Migratory Cells (A) Schematic representation of transwell-based protrusion versus cell-body analysis experiments. (B) Panel of normal and malignant cell lines from diverse tissues of origin, chosen for transwell-based profiling. (C) RP-mRNAs are ubiquitously enriched in protrusions. Transcriptome distributions between protrusion and cell-body fractions were measured by RNA-seq in the panel of cell lines outlined in (B). Log_2_ of protrusion/cell body RNA ratio values for each cell line (Dataset S1) was plotted, with RP-mRNAs highlighted in green. ^∗^MDA- MB231 data were obtained from Mardakheh et al. (2015). All other cell lines were measured from a single matching protrusion and cell-body biological replicate. (D) Validation of RP-mRNA localization to protrusions by RNA-FISH. Representative RNA-FISH images of protrusions and cell bodies of MDA-MB231 cells, stained with probes against the indicated mRNAs (green). Cell boundaries (dashed lines) were defined by co-staining of the cells with anti-tubulin antibody or CellTracker. The filters (gray) were visualized by transmitted light microscopy. (E) Quantification of protrusion to cell-body RNA-FISH ratio values from experiments shown in (D). A total of 6–10 large field of view images from 2 independent experiments were quantified per each probe. (F) Schematic representation of the experimental setting for RNA-FISH imaging of cells invading through 3D collagen-I-matrix. Cells were seeded on the top collagen-I gels and allowed to invade into the matrix for 48 h, before fixation, staining, and confocal imaging of the invaded cells. (G) RP-mRNAs localize to the protrusions of MDA-MB231 cells in 3D. Representative RNA-FISH images of MDA-MB231 cells invading through collagen-I as described in (F), stained with probes against mRNAs (green). Cell boundaries (dashed lines) were defined by co-staining with anti-tubulin antibody. (H) Quantification of the polarization index (PI) values (Park et al., 2012) for the experiments shown in (G), as a measure of displacement of mRNAs away from the cell body. Each data point represents the PI value for a single quantified cell. A total of 22 cells from 2 independent experiments were quantified per each probe. All scale bars, 10 μm.

Previous studies had revealed robust localization of RP-mRNAs to protrusive fronts of mouse NIH-3T3 immortalized fibroblasts (Mili et al., 2008; Wang et al., 2017) and human MDA-MB231 breast cancer cells (Mardakheh et al., 2015). We initially asked whether this localization was restricted to just certain cell types or was a conserved feature of all migratory cells. To systematically profile subcellular mRNA distributions, we utilized a micro-porous transwell-filter-based method (Mardakheh et al., 2015; Mili et al., 2008). We modified the procedure to allow cells to adhere to the top of the filter first, followed by synchronized induction of protrusion formation through the pores (Figure 1A). The small (3 μm) size of the pores enables protrusions to form through the pores but prevents the cell bodies from passing through, thus, resulting in separation of the protrusive fronts and the retractile cell bodies on opposite sides of the filter, which can be independently imaged or purified for multi-omics analysis (Figure 1A). Using this approach, we profiled the subcellular distribution of mRNAs in a diverse panel of normal and malignant migratory human cell lines from various cell types and tissues of origin, by RNA sequencing (RNA-seq) (Figure 1B; Dataset S1). RP-mRNAs were found to be enriched in protrusions of all cell lines (Figures 1C and S1A), strongly supporting the notion that their localization to protrusive fronts is a universal phenomenon.

Next, we validated our RNA-seq results by RNA fluorescence *in situ* hybridization (RNA-FISH). We used specific RNA-FISH probes against five of the top protrusion-enriched RP-mRNAs in the RNA-seq data from MDA-MB231 cells, along with a probe against ITGB4 mRNA as negative control, since it codes for an ER-translated protein and is found to be depleted in protrusions of MDA-MB231 cells by RNA-seq (Dataset S1). All five RP-mRNAs, but not ITGB4 mRNA, were found to be enriched in protrusions of MDA-MB231 cells (Figures 1D, 1E, and S1B). We also validated the protrusion enrichment of three RP-mRNAs in RPE1 cells (Figures S1C and S1D). Next, we assessed the temporal dynamics of RP-mRNAs localization to protrusions. Time-course induction of protrusions followed by RNA-FISH revealed RP-mRNAs enrichment to be persistent for up to at least 8 h (Figures S1E and S1F), suggesting that the localization of RP-mRNAs to protrusions in not a transient phenomenon.

To confirm that the observed enrichment of RP-mRNAs is not restricted to transwell settings, we assessed the localization of RP-mRNAs in actively migrating MDA-MB231 cells. We chose to assess cell migration in 3D as it is more relevant to cell motility *in vivo* (Sahai, 2005) (Figure 1F). RNA-FISH analysis of MDA-MB231 cells invading through a 3D collagen-I matrix revealed RP-mRNAs to be highly enriched at the tip of protrusive fronts, while ITGB4 mRNA remains mostly localized to the perinuclear region (Figures 1G and 1H). Collectively, these results suggest that RP-mRNAs localization to protrusions is a conserved and persistent feature of mesenchymal-like migrating cells.

### Depletion of LARP Proteins Reveals a Role for LARP6 in RP-mRNAs Localization to Protrusions

**Figure 2:**
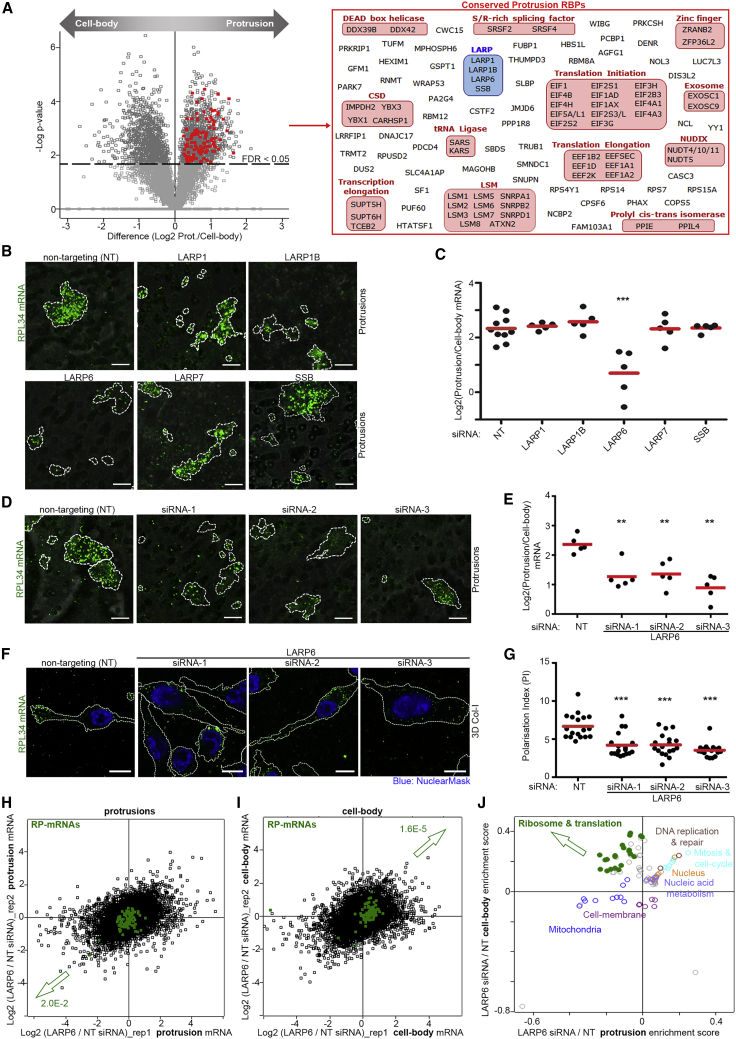
Depletion of LARP Proteins Reveals a Role for LARP6 in RP-mRNAs Localization to Protrusions (A) Quantitative proteomics reveals protrusion-enriched RBPs. Left: volcano plot comparison of protein levels in protrusions relative to cell bodies, across 6 independent cell lines from Figure 1B. Log_2_ of protrusion/cell body protein ratio values from each cell line (Dataset S2) were used to calculate Benjamini-Hochberg corrected p values for protrusion enrichment and depletion, using a one-sample t test analysis. Protrusion-enriched “RNA-binding” proteins (FDR < 0.05), defined according to GOMF database, are marked in red. RIGHT: The list of individual protrusion-enriched RBPs marked on the volcano plot. (B) siRNA screening reveals LARP6 as a crucial regulator of RP-mRNA localization to protrusions. Representative RNA-FISH images of RPL34 mRNA in protrusions of MDA-MB231 cells (green) transfected with non-targeting (NT) control or indicated siRNAs. Cell boundaries (dashed lines) were defined from co-staining with anti-tubulin antibody. The transwell filters (gray) were visualized by transmitted light microscopy. (C) Quantification of RPL34 mRNA enrichment in protrusions from experiments shown in (B). A total of 5–10 large field of view images per condition, measured from 3 independent experiments, were quantified. p values were calculated using two-tailed homoscedastic t test. ^∗∗∗^p < 0.001. (D) Validation of LARP6 by 3 independent siRNAs. Representative RNA-FISH images of RPL34 mRNA in protrusions of MDA-MB231 cells (green) transfected with control or 3 independent LARP6 siRNAs. Cell boundaries (dashed lines) were defined from co-staining with anti-tubulin antibody. The transwell filters (gray) were visualized by transmitted light microscopy. (E) Quantification of RPL34 mRNA enrichment in protrusions from experiments shown in (D). A total of 5 large field of view images per condition, measured from 2 independent experiments, were quantified. p values were calculated using two-tailed homoscedastic t test. ^∗∗^p < 0.01. (F) LARP6 depletion prevents RP-mRNAs localization to protrusions of 3D invading cells. Representative RNA-FISH images of RPL34 mRNA distributions in NT- or LARP6 siRNA-transfected MDA-MB231 cells (green) invading through 3D collagen-I matrix, as described in Figure 1F. Cell boundaries (dashed lines) were defined from co-staining with anti-tubulin antibody. (G) Quantification of the polarization index values from experiments shown in (F) as a measure of displacement of mRNAs away from the cell body. Each data point represents the PI value for a single quantified cell. A total of 18 cells per condition from 2 independent experiments were quantified. p values were calculated using two-tailed, homoscedastic t test. ^∗∗∗^p < 0.001. (H) Depletion of LARP6 significantly reduces RP-mRNA levels in protrusions. MDA-MB231 cells transfected with NT control or LARP6 siRNAs were subjected to transwell fractionation followed by RNA-seq. Log_2_ of NT/LARP6 KD transcript read counts in the protrusion fractions from 2 independent experiments are plotted (Dataset S3), with RP-mRNAs marked in green. Arrow marks the direction of RP-mRNA shift, with the Benjamini-Hochberg-corrected p value of the shift reported next to it. (I) Depletion of LARP6 significantly increases RP-mRNA levels in cell bodies. Log_2_ of NT/LARP6 KD transcript read counts in cell-bodies of the cells described in (H) are plotted (Dataset S3), with RP-mRNAs marked in green. Arrow marks the direction of RP-mRNA shift, with the Benjamini-Hochberg corrected p value of the shift reported next to it. (J) LARP6 depletion induces mis-localization of RP-mRNAs from protrusions to cell bodies. 2D-annotation enrichment analysis (Cox and Mann, 2012) of data shown in (H) and (I). Each data point represents a functional category from GO and KEGG databases, with similar categories being highlighted in the same colors (Dataset S4). Upon LARP6 depletion, mRNAs coding for ribosomal and translation-related categories (green) change in an anti-correlative fashion in protrusions and cell-bodies, suggestive of mis-localization. Other significantly altered categories change in a correlative fashion, suggestive of expression change throughout the cell. All scale bars, 10 μm.

RNA localization is driven by specific RBPs that bind to and mediate transport or anchoring of target transcripts (Eliscovich and Singer, 2017). We therefore hypothesized that specific protrusion-localized RBPs must be interacting with and localizing RP-mRNAs to protrusions. As RP-mRNAs localization was conserved across all the cell lines tested, localizing RBPs must also be conserved across all of them. To reveal conserved protrusion-localized RBPs, we profiled the distribution of proteins between protrusions and cell bodies in our panel of cell lines by tandem mass tagging (TMT)-mediated quantitative proteomics (McAlister et al., 2012) (Dataset S2). We then evaluated which RBPs were significantly enriched in protrusions across the cell lines. 111 RBPs were identified, several of which belong to structurally/functionally related protein categories (Figure 2A). One such category was the La-related proteins, comprised LARP1 and several of its paralogs (Figure 2A). As LARP1 is known to directly bind RP-mRNAs (Al-Ashtal et al., 2019; Fonseca et al., 2015; Hong et al., 2017; Lahr et al., 2017; Tcherkezian et al., 2014), we assessed whether it was important for RP-mRNAs localization to protrusions, using an RNA-FISH probe against RPL34 mRNA, which is one of the most enriched RP-mRNAs in protrusions of MDA-MB231 cells (Figure 1D). LARP1 depletion did not have an impact on RPL34 mRNA localization (Figures 2B and 2C). Furthermore, inhibition of mTORC1 did not affect RPL34 mRNAs localization (Figures S2A–S2C), together suggesting that RPL34 mRNA localization must be independent of the mTORC1-LARP1 pathway.

Next, we depleted other LARP family members that were found to be significantly enriched in protrusions, along with LARP7, which was enriched just below the significance cutoff (Dataset S2). Only the depletion of LARP6 resulted in a significant decrease in localization of RPL34 mRNA to protrusions (Figures 2B and 2C). This decrease was reproduced by 3 independent siRNAs (Figures 2D, 2E, and S2D), without having an impact on the ability of cells to form protrusions per se (Figure S2E), and could be rescued by stable expression of an siRNA-resistant GFP-tagged LARP6 construct (Figures S2F and S2G). Localization of RPL34 mRNAs in 3D invading MDA-MB231 cells was also significantly affected upon LARP6 depletion (Figures 2F and 2G). Moreover, CRISPR-Cas9-mediated knockout (KO) of LARP6 similarly reduced RPL34 mRNA localization to protrusions (Figures S2H–S2J). Finally, short-term (2 h) treatment of protruding cells with C9, a small-molecule inhibitor that specifically interferes with LARP6 RNA binding (Stefanovic et al., 2019), also reduced RPL34 mRNA localization (Figures S2K and S2L). Together, these results robustly demonstrate that LARP6 localizes RPL34 mRNA to protrusions.

To confirm that the impact of LARP6 depletion was not restricted to just one RP-mRNA, we carried out RNA-seq analysis of protrusion and cell-body fractions from control and LARP6 knockdown cells. Depletion of LARP6 resulted in a significant decrease in the overall levels of RP-mRNAs in protrusions, along with a concomitant increase in their levels within the cell bodies (Figures 2H–2J; Datasets S3 and S4). Accordingly, the relative enrichment of RP-mRNAs in protrusions was lost in LARP6-depleted cells (Figure S2M). Collectively, these results suggest that LARP6 is critical for localization of RP-mRNAs to protrusions.

### Transcriptome-Wide iCLIP Studies Reveal Direct Binding of LARP6 to RP-mRNAs

**Figure 3:**
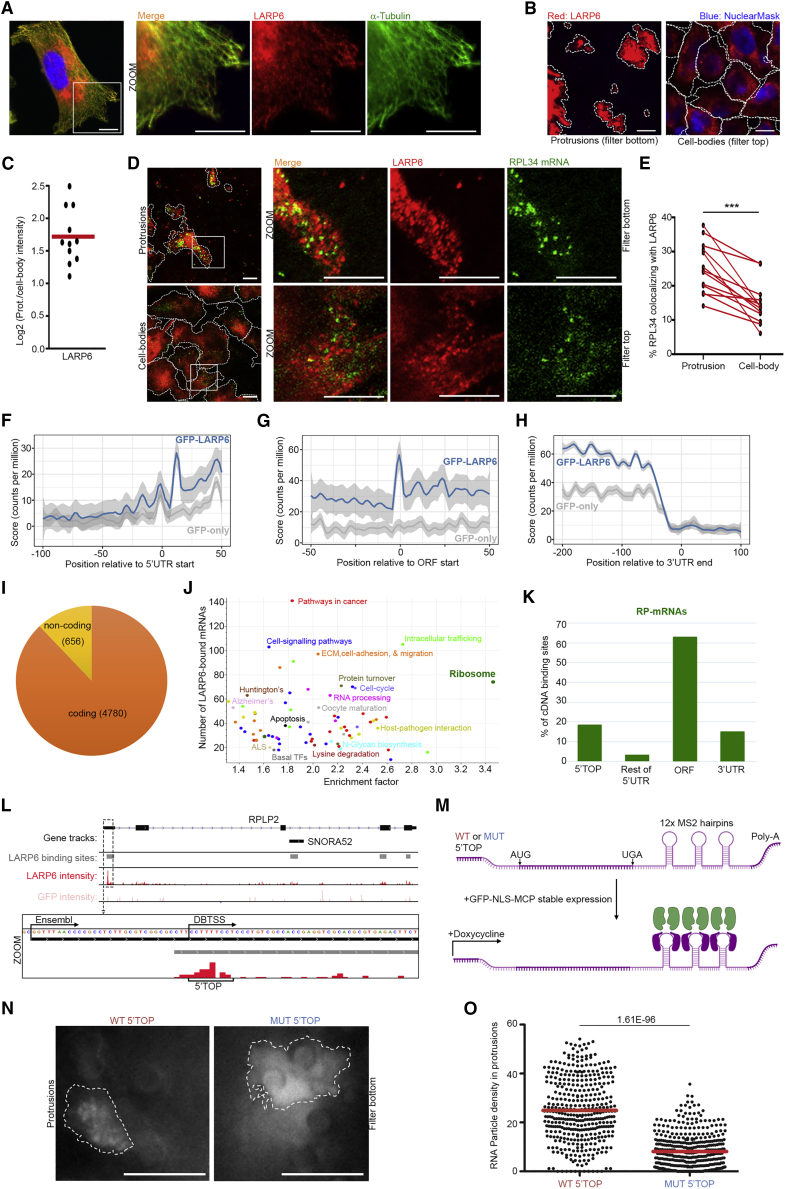
Transcriptome-wide iCLIP Studies Reveal Direct Binding of LARP6 to RP-mRNAs (A) LARP6 is localized to cytoplasmic puncta that track microtubules. Representative IF images of LARP6 (red) and α-tubulin (green) in MDA-MB231 cells grown on collagen-coated slides. Nucleus was stained with NuclearMask (blue). (B) LARP6 puncta are enriched in protrusions. Representative IF images of LARP6 (red) in protrusions and cell bodies of MDA-MB231 cells. Cell boundaries (dashed lines) were defined from co-staining with anti-tubulin antibody. (C) Quantification of IF images from experiments shown in (B), revealing LARP6 enrichment in protrusions. A total of 11 large field of view images, measured from 2 independent experiments were quantified. (D) LARP6 co-localizes with RP-mRNAs in protrusions. Representative RNA-FISH and IF co-staining images of RPL34 mRNA (green) and LARP6 (red) in protrusions and cell bodies of MDA-MB231 cells. Cell boundaries (dashed lines) were defined from co-staining with anti-tubulin antibody. (E) Quantification of the % of co-localization of RPL34 mRNA with LARP6 in corresponding protrusion and cell-body images from experiments shown in (D). A total of 13 large field of view images from 2 independent experiments were quantified. Red lines connect values of protrusion and body from the corresponding images. p values were calculated using a two-tailed, homoscedastic t test. ^∗∗∗^p < 0.001. (F) Metaprofile plot of LARP6 iCLIP crosslink sites at the aligned annotated intergenic-5′UTR junctions (2,204 landmarks), showing preferential association with specific regions at the vicinity of TSS. (G) Metaprofile plot of LARP6 iCLIP crosslink sites at the aligned annotated 5′UTR-ORF junctions (4,122 landmarks), showing preferential association with the translation start site. (H) Metaprofile plot of LARP6 iCLIP crosslink sites at the aligned annotated 3′UTR-intergenic junctions (6,333 landmarks), showing association throughout the 3′UTR. (I) LARP6 mainly binds protein-coding transcripts. Pie chart showing the prevalence of coding versus non-coding RNAs among LARP6 binding targets (Dataset S6). (J) The KEGG category of ribosome (green), which is comprised all RP-mRNAs, is significantly enriched among LARP6-binding targets. Fisher’s exact test analysis (FDR < 0.02) of mRNA categories, which are significantly over-represented among the identified LARP6 targets. Each data point represents a functional category from KEGG database, with similar categories highlighted by the same colors (Dataset S7). (K) LARP6 interacts with RP-mRNAs via multiple regions. Distribution of LARP6-binding regions in RP-mRNAs. (L) An example genomic view of LARP6-specific binding sites after peak calling (gray tracks) in an RP-mRNA (RPLP2), along with read intensities for GFP and GFP-LARP6 iCLIP runs. Four distinct LARP6-binding sites are mapped to the RPLP2 locus: two mapping to the ORF region, one to RPLP2 3rd intron, which is annotated as SNORA52, and one to the 5′UTR. Inset: zoomed view of RPLP2 5′UTR showing the LARP6-binding site overlapping with the 5′TOP. Note that for most RP-mRNAs, annotation of TSS in Ensembl is further upstream of the more accurately annotated DBTSS (Suzuki et al., 2018). (M) Schematic representation of the MS2 reporter system for live-cell monitoring of 5′TOP mediated RNA localizations. (N) WT 5′TOP motif is sufficient for RP-mRNA localization to protrusions. Representative still images of the GFP-MCP signal in transwell protrusions of WT or MUT 5′TOP reporter engineered MDA-MB231 cells described in (M), following induction of reporter expression with 2 μg/mL doxycycline for 12 h. GFP-MCP exhibits a punctate pattern in protrusions of WT 5′TOP reporter expressing cells, indicative of association with mRNA particles, as opposed to a diffuse pattern in protrusion of MUT 5′TOP reporter expressing cells. (O) Quantification of mRNA particles in protrusions of WT-5’TOP versus MUT-5’TOP reporter expressing cells from experiments shown in (N). A total of 25 (WT) and 28 (MUT) time-lapse videos (3 s at 0.2-s intervals) from 2 independent experiments were quantified. The number of discrete particles identified at every frame image were quantified and normalized to the protrusion area to determine mRNA molecule density. The p value was calculated using a two-tailed, homoscedastic t test. All scale bars, 10 μm.

We next investigated the localization and function of LARP6. To study the subcellular localization of LARP6, we used immunofluorescence (IF) with a specific antibody against LARP6 (Figures S3A and S3B). LARP6 exhibits a cytoplasmic punctate localization, with LARP6 puncta closely tracking the microtubule filaments (Figures 3A and S3C), a feature consistent with an RBP that functions in RNA localization (Bullock, 2011). In agreement with proteomics enrichment of LARP6 in protrusions (Figure S3D), IF analysis revealed LARP6 to be highly enriched in protrusions (Figures 3B and 3C). Furthermore, a fraction of RPL34 mRNA co-localizes with LARP6, with the co-localization being significantly enhanced in protrusions (Figures 3D and 3E).

Given the co-localization of RP-mRNAs with LARP6 in protrusions, we wished to determine whether they directly interact. Collagen type I alpha-1 and alpha-2 (COL1A1 and COL1A2) mRNAs have so far been the only known RNA-binding partners of LARP6 (Cai et al., 2010; Martino et al., 2015). However, COL1A1 and COL1A2 mRNAs were enriched in the cell bodies of the many cell lines we examined (Figure S3E), indicating that other mRNA partners are likely to be relevant for the LARP6 function in protrusions. In order to identify direct RNA-binding sites of LARP6 across the transcriptome, we utilized MDA-MB231 cells that stably express GFP-tagged LARP6 or GFP alone as control and performed individual-nucleotide resolution UV crosslinking and immunoprecipitation (iCLIP) by anti-GFP beads (König et al., 2010). Comparison of crosslink read counts between GFP and GFP-LARP6 immunoprecipitates revealed a clear LARP6-dependent enrichment (Figure S3F), confirming iCLIP specificity. In agreement with LARP6 cytoplasmic localization, its crosslinking was strongly enriched on exonic compared with intronic regions (Figure S3G). Among mRNAs, crosslinking on 3′UTRs was 2–3-fold higher compared with 5′UTR and open reading frame (ORF) sequences (Figure S3G). Analysis of crosslink sites at aligned 5′UTR sequences revealed spikes of LARP6-specific crosslinks at the vicinity of the transcription start site (TSS) (Figure 3F). A clear spike of LARP6 specific crosslinks was also observed at the translation start site (Figure 3G), while no apparent positional bias was evident in distribution of LARP6 crosslinks at the 3′UTR (Figure 3H).

Next, we searched for clusters of crosslinking across the genome, which identified peaks corresponding to likely binding sites. A total of 5,135 peaks were detected for GFP, whereas 21,094 peaks were identified for GFP-LARP6. Of these, 2,704 overlapped with GFP peaks, while 18,390 were unique, corresponding to likely LARP6-binding sites (Dataset S5). These peaks mapped to a total of 5,436 genes (Dataset S6), the vast majority of which were protein coding (Figure 3I). Enrichment analysis revealed RP-coding transcripts (i.e., RP-mRNAs) as the most enriched mRNA category (Figure 3J; Dataset S7), with LARP6 binding sites found in 73 RP-mRNAs (Figure S3H). Other significantly enriched categories included transcripts involved in RNA processing, intracellular trafficking, cell migration, adhesion, and extracellular matrix (ECM), among others (Figure 3J; Dataset S7). Such diversity in targets is in line with recent *in vitro* findings that have revealed LARP6 to possess a highly complex binding specificity, capable of interacting with multiple structural as well as short or gapped linear motifs (Jolma et al., 2020). Together, these results reveal that LARP6 binds to a plethora of transcripts, with RP-mRNAs constituting one of the major target groups.

We then investigated the mechanism of LARP6 binding and regulation of RP-mRNAs. Around 60% of LARP6 peaks within RP-mRNAs were located in the ORF, with the remaining peaks mainly mapping to the 5′TOP motif, followed by the 3′UTR, and a minor portion to regions downstream of 5′TOP in the 5′UTR (Figure 3K). The majority of RP-mRNAs contained two or more LARP6-binding sites (Figures 3L and S3H). We also detected LARP6 peaks within introns of 43 RP genes, but the majority of these overlapped with 37 annotated small nucleolar RNAs (SNORs) that are encoded within these introns (Figure 3L). The positioning of these peaks indicates that LARP6 also binds to SNORs that are processed from the introns of RP-mRNAs.

As the 5′TOP motif is conserved across all RP-mRNAs, we next investigated whether this motif alone could be sufficient for localizing mRNAs to protrusions. We used an MS2 based live-cell RNA imaging system (Bertrand et al., 1998) to visualize the subcellular localization of reporter mRNAs that contain either a wild-type (WT) or a mutant (MUT) 5′TOP motif (Gentilella et al., 2017) (Figure 3M). We first validated the inducible expression of both reporter constructs in our cells by live-cell imaging (Figures S3I and S3J; Videos S1, S2, S3, and S4). Using transwell filters, we then assessed the localization of reporter mRNA particles to protrusion. While WT 5′TOP containing mRNA particles readily traveled to protrusions, MUT 5′TOP containing mRNA particles were rarely detectable in protrusions (Figures 3N and 3O; Videos S5 and S6). Together, these results reveal that harboring a single 5′TOP motif is sufficient to target mRNAs to protrusions.

Video S1. Time-Lapse Analysis of WT 5′TOP MS2 Reporter MDA-MB231 Cells Without Doxycycline, Related to Figure 3 200-ms frame images were taken for 10 s at 100× magnification, showing diffuse localization of MCP-GFP. Results are representative of 3 independent experiments.

Video S2. Time-Lapse Analysis of WT 5′TOP MS2 Reporter MDA-MB231 Cells with Doxycycline (2 μg/mL for 12 h), Related to Figure 3 200-ms frame images were taken for 10 s at 100× magnification, showing MCP-GFP bound cytoplasmic WT 5′TOP mRNA particles. Results are representative of 3 independent experiments.

Video S3. Time-Lapse Analysis of MUT 5′TOP MS2 Reporter MDA-MB231 Cells Without Doxycycline, Related to Figure 3 200 ms frame images were taken for 10 s at 100× magnification, showing diffuse localization of MCP-GFP. Results are representative of 3 independent experiments.

Video S4. Time-Lapse Analysis of MUT 5′TOP MS2 Reporter MDA-MB231 Cells with Doxycycline (2 μg/mL for 12 h), Related to Figure 3 200 ms frame images were taken for 10 s at 100× magnification, showing MCP-GFP bound cytoplasmic MUT 5′TOP mRNA particles. Results are representative of 3 independent experiments.

Video S5. Time-Lapse Analysis of Protrusions of WT 5′TOP MS2 Reporter MDA-MB231 Cells with Doxycycline (2 μg/mL for 12 h), Related to Figure 3 Cells were grown on transwell filters and induced to form protrusions for 2 h prior to imaging. 200 ms frame images were taken for 3 s at 100× magnification from the bottom of transwell filters, showing MCP-GFP bound WT 5′TOP mRNA particles within protrusions. Results are representative of 2 independent experiments.

Video S6. Time-Lapse Analysis of Protrusions of MUT 5′TOP MS2 Reporter MDA-MB231 Cells with Doxycycline (2 μg/mL for 12 h), Related to Figure 3 Cells were grown on transwell filters and induced to form protrusions for 2 h prior to imaging. 200 ms frame images were taken for 3 s at 100× magnification from the bottom of transwell filters, showing no MCP-GFP bound MUT 5′TOP mRNA particles within protrusions. Results are representative of 2 independent experiments.

### LARP6-Dependent RP-mRNA Localization Enhances RP Synthesis and Ribosome Biogenesis

**Figure 4:**
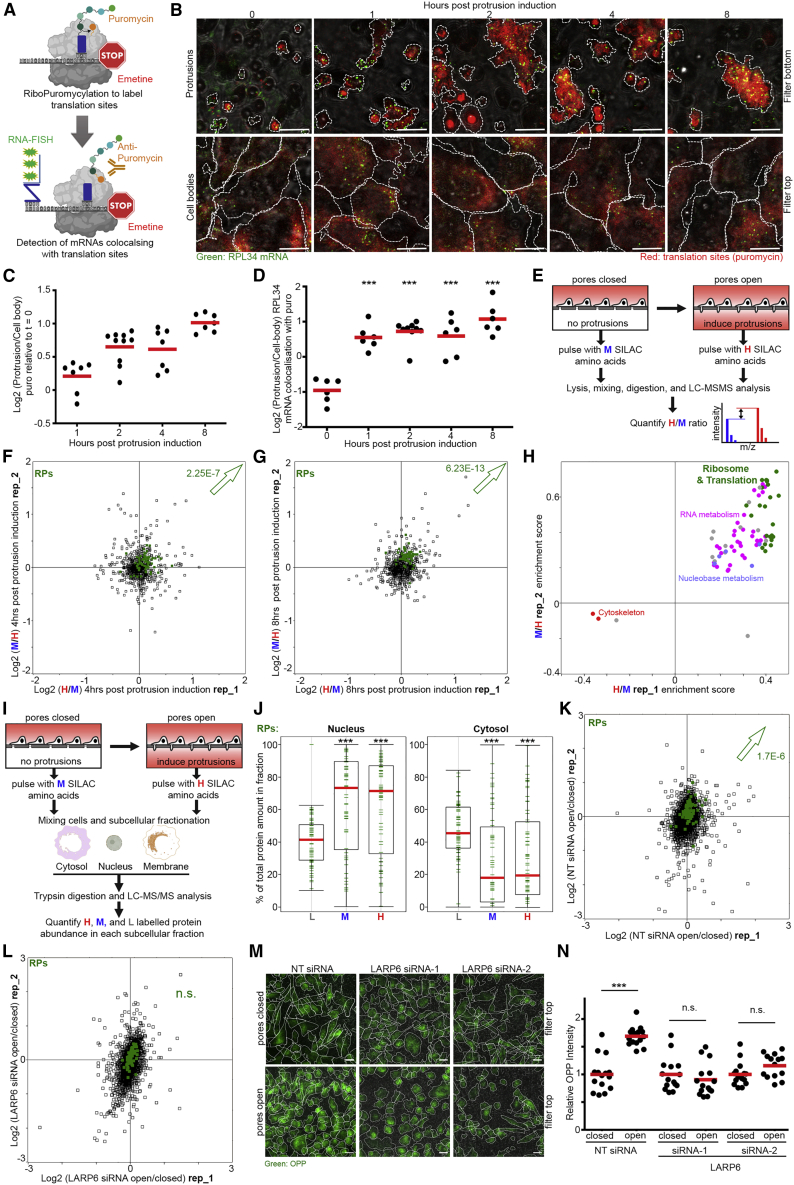
LARP6-Dependent RP-mRNA Localization Enhances RP Synthesis and Ribosome Biogenesis (A) Schematic representation of the Ribopuro-FISH assay. A short pulse of puromycin results in labeling of nascent proteins. When emetine is present, puromycylated peptides remain associated to the ribosome. Detection of these peptides with anti-puromycin antibody visualizes cellular sites of active translation. Co-detection of a specific mRNA by RNA-FISH marks the fraction of mRNA associated with translation sites. (B) RP-mRNAs are associated with active sites of translation in protrusions. Representative Ribopuro-FISH images of RPL34 mRNA (green) and puromycin (red) in protrusions and cell bodies of MDA-MB231 cells at the indicated time points post protrusion induction. Cell boundaries (dashed lines) were defined from co-staining with anti-tubulin antibody. All scale bars, 10 μm. (C) Translation in protrusions relative to the cell bodies increases over time. Quantification of puromycin staining intensities in protrusions relative to cell bodies, from experiments shown in (B). A total of 7–10 large field of view images per condition, from 2 independent experiments, were quantified. (D) Association of RPL34 mRNAs with active sites of translation is higher in protrusions than cell bodies. Quantification of % RPL34 mRNA co-localization with puromycin in protrusions relative to the cell bodies from experiments shown in (B). A total of 6–10 large field of view images per condition were quantified as in (C). p values were calculated for each time-point relative to time zero, using a two-tailed, homoscedastic t test. ^∗∗∗^p < 0.001. (E) Schematic diagram of pulsed SILAC proteomics analysis of changes in protein translation rates induced by protrusion formation. Light (L) SILAC-labeled MDA-MB231 cells were grown overnight on top of two transwell filters without any media in the bottom chamber. The next day, media on top was changed to medium (M) or heavy (H) SILAC media, followed by addition of the same label media to the bottom chamber of one of the two transwells in order to open the pores to the cells. Cells were then allowed to form protrusions for 1, 2, 4, or 8 h, or left without protrusions for the same length of time as control. H/M ratios for each protein were determined by MS analysis of the whole cell lysates, as measurement of translation rate changes between open pore (with protrusions) and closed pore (without protrusions) conditions (Dataset S9). (F) Translation of RPs (green) is significantly increased after 4 h of protrusion formation. Log_2_ of H/M ratio values from 2 reciprocally labeled biological replicate experiments were plotted against each other (Dataset S9). Arrow marks the direction of shift in RPs, with Benjamini-Hochberg corrected p value of the shift reported next to it. (G) Translation of RPs (green) is significantly increased after 8 h of protrusion formation. Log_2_ of H/M ratio values from 2 reciprocally labeled biological replicate experiments were plotted against each other (Dataset S9). Arrow marks the direction of shift in RPs, with Benjamini-Hochberg corrected p value of the shift reported next to it. (H) 2D-annotation enrichment analysis of data shown in (F) and (G). Each data point represents a functional category from GO and KEGG databases, with similar categories highlighted with the same colors (Dataset S10). Translation of ribosomal and translation-related protein categories (green), as well as a number of RNA-metabolism-related protein categories (pink), is significantly enhanced following protrusion induction for 4 and 8 h. (I) Schematic representation of the experimental outline for pulsed SILAC mediated assessment of subcellular distributions of nascent proteins following protrusion induction. Absolute abundances of light (L)-, medium (M)-, and heavy (H)-labeled proteins in each subcellular compartment were measured by iBAQ, in presence or absence of protrusions, and used to calculate the % of labeled protein in each compartment. (J) Newly synthesized RPs accumulate in the nucleus. Box plot of the % of old and nascent RPs in the nuclear and cytosolic fractions of MDA-MB231 cells. Old RPs (L), nascent RPs synthesized under basal conditions without protrusions (M), and nascent RPs synthesized under protrusion-induced condition (H) were distinguished by their SILAC labeling state and separately quantified in each fraction within a single experiment (Dataset S11). Error bars are min-max range. Significance p values were calculated using a two-way t test analysis between L and M or H values. ^∗∗∗^p < 0.001. (K) Total RP levels are significantly increased upon long-term protrusion induction in NT control siRNA-treated MDA-MB231 cells. Proteome changes between closed and open-pore (overnight) conditions in NT control siRNA-treated MDA-MB231 cells were quantified by TMT quantitative proteomics (Dataset S14). Log_2_ of NT siRNA open/close ratios from 2 biological replicate experiments were plotted against each other. The arrow marks the direction of shift in RP levels, with Benjamini-Hochberg corrected p value of the shift reported next to it. (L) Total RP levels are do not significantly change upon long-term protrusion induction in LARP6 siRNA-treated MDA-MB231 cells. Proteome changes between closed and open pore (overnight)-conditions in LARP6 siRNA-treated MDA-MB231 cells were quantified by TMT quantitative proteomics (Dataset S14) (n.s., not significant). Log_2_ of LARP6 siRNA open/close ratios from 2 biological replicate experiments were plotted against each other. (M) LARP6 depletion inhibits protrusion-induced enhancement of overall protein synthesis. Transwell seeded NT control and 2 independent LARP6 siRNA-treated MDA-MB231 cells were either prevented from protruding through pores (pores closed), or allowed to form protrusions (pores open) for 24 h, before labeling with OPP for 15 min. OPP was then visualized by Click-chemistry-mediated Alexa Fluor-488 labeling. Representative images of the cells from top of the filters are displayed. Cell boundaries (dash lines) were defined by anti-tubulin staining. Scale bars, 20 μm. (N) Quantification of normalized OPP staining levels from (M). A total of 15 large field of view images per condition from 2 independent experiments were quantified. p values were calculated using two-tailed, homoscedastic t test. n.s., non-significant; ^∗∗∗^p < 0.001.

Next, we investigated the functional consequence of RP-mRNA targeting to the protrusive fronts by LARP6. Our profiling of protein distributions between protrusions and cell bodies had revealed many translation initiation and elongation factors as enriched in protrusions (Figure 2A; Dataset S2). In fact, time-course analysis of the proteome distribution between protrusions and cell bodies of MDA-MB231 cells showed that proteins involved in translational initiation and elongation accumulate in protrusions early on and remain localized (Figures S4A and S4B; Dataset S8). We therefore hypothesized that this enrichment could lead to higher local levels of translation, making protrusions function as hotspots for translation of localized transcripts. To assess this hypothesis, we mapped the subcellular distribution of translation sites in MDA-MB231 cells using RiboPuromycylation (Bastide et al., 2018). We optimized the RiboPuromycylation method so that it could be used concurrently with RNA-FISH (Figure 4A), thus, allowing the investigation of whether an RNA of interest is associated with translation sites at a given location. In agreement with the observed accumulation of translation initiation and elongation factors in protrusions, time-course RiboPuromycylation analysis of transwell protruding MDA-MB231 cells revealed translation sites to be enriched in protrusions (Figures 4B and 4C). Moreover, co-localization of RP-mRNAs with translation sites was significantly higher in protrusions than the cell bodies (Figures 4B and 4D), suggesting that protrusion-localized RP-mRNAs are likely to undergo more translation. Indeed, using a pulsed stable isotope labeling of amino acids in cell culture (pulsed SILAC; Schwanhäusser et al., 2009)-based strategy (Figure 4E), we could show that overall translation of RPs was significantly enhanced after allowing cells to form protrusions for 4 or 8 h (Figures 4F–4H; Datasets S9 and S10). These results demonstrate that protrusion formation acts to enhance the overall translation of RPs.

Local translation of RP-mRNAs might increase RP abundance just in protrusive fronts. Alternatively, newly made RPs might translocate into the nucleus in order to interact with maturing rRNAs and contribute to ribosome biogenesis (Bohnsack and Bohnsack, 2019). To distinguish between these two possibilities, we combined our pulsed SILAC strategy with subcellular fractionation of cells into nuclear, membrane, and cytosolic fractions (Figures 4I and S4C). Overall, RPs were mostly found to reside in the cytosol and nucleus, but not the membrane fraction (Dataset S11). Newly synthesized RPs, from both with (open pores) and without (closed pores) protrusion conditions, showed a strong accumulation in the nucleus (Figure 4J; Dataset S11). In contrast, pre-existing RPs that constitute RPs in mature ribosomes accumulated more in the cytosol (Figure 4J; Dataset S11). These results indicate that similar to the basally translated nascent RPs, most protrusion-synthesized nascent RPs translocate to the nucleus to participate in canonical ribosome biogenesis.

While augmented translation of RP-mRNAs is necessary for increased ribosome biogenesis, newly synthesized RPs are normally degraded in the nucleus if not incorporated into new ribosomes (Lam et al., 2007). We therefore wanted to test whether enhanced translation of RPs upon protrusion induction does indeed result in higher total levels of RPs. TMT-mediated quantitative proteomics revealed that while short (2 h) induction of protrusions did not significantly change total RP levels, a longer (24 h) induction resulted in a modest yet significant increase in RP levels (Figures S4D and S4E; Datasets S12 and S13). Accordingly, O-propargyl-puromycin (OPP) labeling, a method to measure protein synthesis by incorporation of an alkyne analog of puromycin (Liu et al., 2012), revealed a significant boost in the overall protein synthesis following longer protrusion induction that is in agreement with increased ribosome biogenesis (Figures S4F and S4G).

We next tested whether the observed increase in ribosome biogenesis following longer protrusion induction is LARP6 dependent. While total RP levels were upregulated following protrusion induction in non-targeting control siRNA-treated cells (Figure 4K; Dataset S14), no significant increase was observed in LARP6 siRNA-treated cells (Figure 4L; Dataset S14). In addition, enhancement of overall protein synthesis upon protrusion induction was inhibited by LARP6 depletion (Figures 4M and 4N). Together, these results demonstrate that upon protrusion formation, LARP6-dependent localization of RP-mRNAs promotes their translation, ultimately leading to enhanced ribosome biogenesis and upregulated overall protein synthesis.

### LARP6 Is Important for Ribosome Biogenesis, Invasion, and Proliferation of Migrating Cells

**Figure 5:**
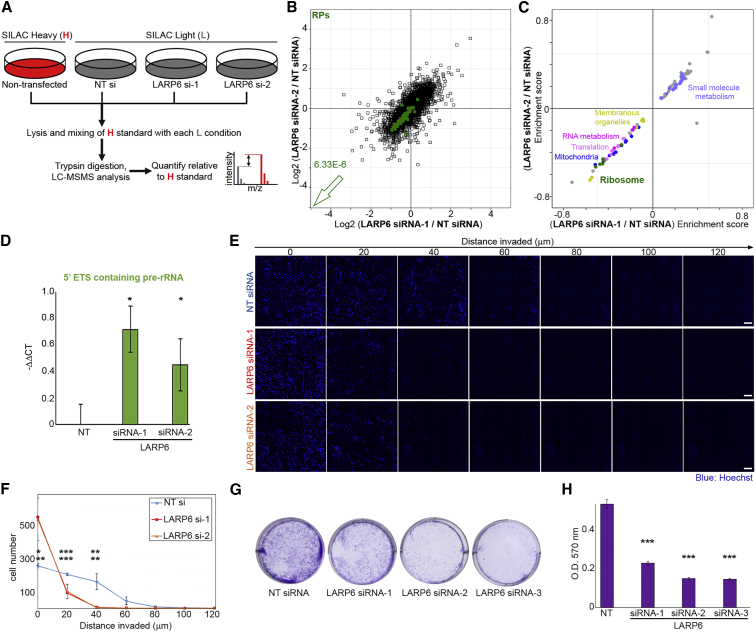
LARP6 Is Important for Ribosome Biogenesis, 3D Invasion, and Proliferation of Migrating Cells (A) Schematic representation of SILAC proteome analysis following LARP6 depletion. Light (L) SILAC-labeled MDA-MB231 cells, transfected with NT control siRNA or 2 independent LARP6 siRNAs for 72 h, were lysed and mixed with H-labeled non-transfected MDA-MB231 lysates as reference. H/L ratio values in each mix was then used to calculate relative protein abundance changes. (B) LARP6 depletion significantly decreases total RP levels in MDA-MB231 cells. Changes in individual protein levels following LARP6 depletion with 2 independent siRNAs were quantified as described in (A) and plotted (Dataset S15). Benjamini-Hochberg corrected p value of decrease in RP (green) levels is reported on the graph. (C) 2D-annotation enrichment analysis of data shown in (B). Each data point represents a protein category inferred from GO and KEGG, and similar categories are highlighted by the same colors (Dataset S16). Categories of proteins comprised RPs (green), translation related (light pink), and RNA metabolism related (pink) are all significantly downregulated upon LARP6 depletion by 2 independent siRNAs. (D) LARP6 depletion results in accumulation of 5′ETS containing pre-rRNAs. RT-qPCR of 5′ETS pre-rRNA in MDA-MB231 cells transfected with NT control siRNA or 2 independent LARP6 siRNAs for 72 h. A specific probe against the 5′ETS region, along with a specific probe against GAPDH mRNA as loading control, were used to quantify –ΔΔCT values. Average values were calculated from 3 independent experiments, each performed in at least 3 technical replicates, per condition. Error bars are SD. p values were calculated using two-tailed, homoscedastic t test. ^∗^p < 0.05. (E) LARP6 depletion hampers the ability of MDA-MB231 cells to invade through 3D collagen. MDA-MB231 cells were treated with NT control siRNA or 2 independent siRNAs against LARP6 for 72 h before being subjected to 3D collagen-I Invasion assay. 5 × 5 tiled confocal images of fixed, Hoechst-stained cells (blue) at different migrated distances from the start point are displayed. Scale bars, 200 μm. (F) Quantification of invaded cell numbers from (E). Average values were calculated from 3–5 biological replicates per condition. Error bars are SD. p values were calculated using two-tailed, homoscedastic t test. ^∗^p < 0.05; ^∗∗^p < 0.01; ^∗∗∗^p < 0.001. (G) Long-term LARP6 depletion decreases MDA-MB231 proliferation. MDA-MB231 cells were transfected with NT control siRNA or 2 independent LARP6 siRNAs for 72 h, before reseeding to form colonies for a further 10 days prior to crystal violet staining. (H) Optical density of crystal-violet-stained colonies from experiments shown in (G) were measured by 570-nm absorbance (*OD*_*570*_) after dye extraction. Average values were calculated from 3 independent experiments, each performed in 3 technical replicates. Error bars are SD. p values were calculated using two-tailed, homoscedastic t test. ^∗∗∗^p < 0.001.

Since our findings above provide a link between cell migration and regulation of ribosome biogenesis, we next investigated whether LARP6 contributes toward a significant proportion of RP synthesis in migratory mesenchymal-like cells. Using SILAC, we quantified the impact of LARP6 depletion on the proteome of actively growing MDA-MB231 cells (Figure 5A). RPs were significantly decreased upon LARP6 knockdown (Figure 5B; Dataset S15). In fact, category enrichment analysis revealed that RPs were among the most downregulated protein categories following LARP6 depletion (Figure 5C; Dataset S16). As availability of RPs is crucial for processing and maturation of rRNA during ribosome biogenesis, a substantial decrease in their expression would result in accumulation of otherwise transient pre-rRNA transcripts, which can be detected by RT-qPCR (Piñeiro et al., 2018). Accordingly, LARP6 knockdown resulted in a significant accumulation of pre-rRNAs that contain the 5′ external transcribed spacer (5′ETS) (Figure 5D), suggesting that the decrease in total RP levels due to LARP6 depletion must be significant enough to hamper rRNA processing.

Increased ribosome biogenesis underpins various aspects of cellular life such as enhanced proliferation, migration, and invasion (Pelletier et al., 2018). We therefore assessed whether depletion of LARP6 compromised proliferation and 3D migration of MDA-MB231 cells. Indeed, LARP6 knockdown by two independent siRNAs significantly reduced the ability of MDA-MB231 cells to invade through 3D Collagen (Figures 5E and 5F). Knockdown of LARP6 also decreased the viability of MDA-MB231 cells, but this decrease was only significant after longer-term depletion of LARP6 (Figure S5A), suggesting that the observed decrease in invasiveness is unlikely to be an indirect consequence of viability loss. Accordingly, LARP6 knockdown significantly affected the long-term growth of MDA-MB231 cells as revealed by clonogenic assays (Figures 5G and 5H). Interestingly, CRISPR-Cas9 KO clones of LARP6 are viable and only mildly, albeit still significantly, affected by loss of LARP6 (Figure S5B). As cells undergo long-term selection during isolation of outgrowing single CRISPR-Cas9 clones, it is possible that other mechanisms of RP synthesis that compensate for loss of LARP6 have been positively selected for in our KO cells. Recently described transcriptional compensation mechanisms triggered by CRISPR-Cas9 but not RNAi may also be at play (El-Brolosy et al., 2019; Ma et al., 2019). Nevertheless, low doses of C9 treatment could strongly compromise the viability of LARP6 WT but not KO cells (Figure S5C), suggesting that while WT cells are dependent on LARP6 function for their survival, KO cells have acquired LARP6-independent compensatory mechanisms. Together, these results suggest that in mesenchymal-like migratory cells, LARP6-dependent upregulation of ribosome biogenesis plays a crucial role in supporting proliferation and invasion.

### Expression of LARP6 in Cancer Is Triggered by EMT and Acts to Enhance Protein Synthesis

**Figure 6:**
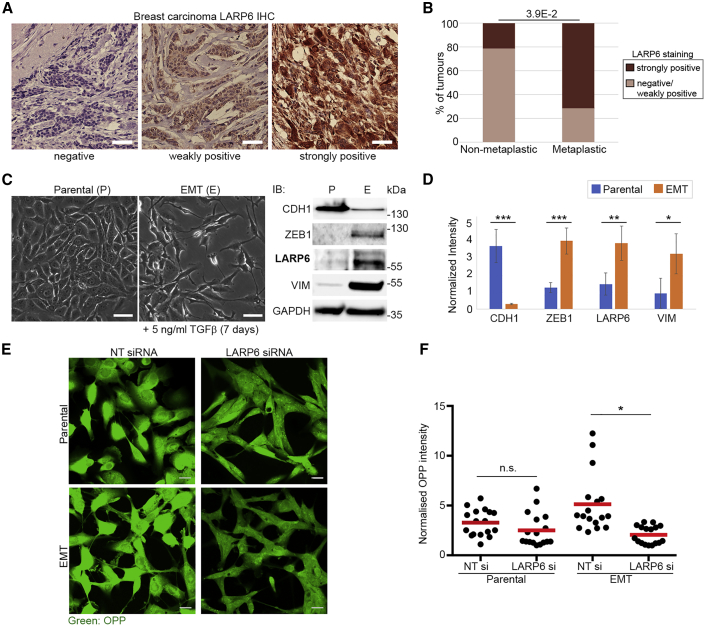
Expression of LARP6 in Cancer Is Triggered by EMT and Acts to Enhance Protein Synthesis (A) Analysis of LARP6 expression in a panel of 33 human breast tumors by IHC. Three distinct patterns of LARP6 expression were detected among the tumor samples: “negative,” “weakly positive,” and “strongly positive.” Representative images for each category are shown. Scale bars, 50 μm. (B) LARP6 strongly positive tumors are significantly enriched among metaplastic carcinomas. Categorizing tumors based on their LARP6 IHC staining status as in (A) reveals a significant enrichment of LARP6 strongly positive tumors among metaplastic carcinomas (n = 7 out of 33). The p value was calculated using Fisher’s exact test. (C) Induction of EMT by human TGF-β1 upregulates LARP6. Left, morphology of MCF10AT cells following mock treatment or TGF-β1 (5 ng/mL) treatment for 7 days, reveals EMT induction. Scale bars, 50 μm. Right, immunoblot (IB) analysis of EMT markers (CDH1, ZEB1, and VIM) and LARP6, on the cells shown in left. GAPDH was used as loading control. (D) Quantification of changes in LARP6 and EMT marker proteins relative to GAPDH, from experiments shown in (C). IBs from 4 independent experiments as in (C) were quantified. Error bars are SD. p values were calculated using two-tailed, homoscedastic t test. ^∗∗∗^p < 0.001; ^∗∗^p < 0.01; ^∗^p < 0.05. (E) EMT enhances overall protein synthesis in a LARP6-dependent manner. MCF10AT parental and EMT pairs from (C) were treated with indicated siRNAs for 72 h before being subjected to OPP staining. (F) Quantification of OPP staining from experiments shown in (D). Normalized OPP averages were calculated from 7–11 field of view images from two independent experiments. Error bars are SD. p values were calculated using two-tailed, homoscedastic t test. n.s., non-significant; ^∗^p < 0.05.

Since enhanced ribosome biogenesis is a common feature of most high-grade carcinomas, we wondered whether the LARP6-dependent RP synthesis could be commonly upregulated in such cancers in order to boost ribosome biogenesis. Mining a published proteomics dataset of protein expression levels in a panel of human breast carcinoma cell lines (Lawrence et al., 2015) revealed LARP6 protein expression to be mainly detectable in cell lines belonging to the mesenchymal/low Claudin subtype (Figure S6A). Similarly, analysis of publicly available mRNA expression data from 1,758 human primary breast tumors (Cerami et al., 2012; Curtis et al., 2012) revealed a significant upregulation of LARP6 in tumors of the mesenchymal/low Claudin subtype (Figure S6B). This molecular subtype is closely associated with EMT and is primarily featured in metaplastic breast carcinomas, a rare but highly invasive form of breast cancer with poor prognosis (Taube et al., 2010). Indeed, immunohistochemistry (IHC) profiling of a panel of human breast tumor tissue samples composed of both metaplastic and non-metaplastic carcinomas revealed a significant association of high LARP6 expression with metaplastic tumors (Figures 6A and 6B).

We next investigated whether the expression of LARP6 protein was directly regulated by EMT. *In vitro*, EMT can be induced by long-term TGF-β1 treatment or forced expression of transcription factors such as Snail or Twist, which act as master inducers of EMT (Taube et al., 2010). Triggering EMT in transformed epithelial-like MCF10AT1 cells by any of these methods resulted in upregulation of LARP6 (Figures 6C, 6D, S6C, and S6D), suggesting that LARP6 expression is directly triggered by and associated with EMT.

Due to the disproportionate upregulation of ribosome biogenesis in most high-grade cancers, there has been a great interest in developing novel strategies that can therapeutically target this pathway in clinic (Pelletier et al., 2018). We hypothesized that in cancers with strong EMT features, inhibiting LARP6 could provide a therapeutic opportunity to more specifically target ribosome biogenesis. In support of this view, induction of EMT in epithelial-like MCF10AT cells enhanced overall protein synthesis in a LARP6-dependent manner (Figures 6E and 6F). Accordingly, while the viability of parental epithelial-like MCF10AT cells was only mildly affected by LARP6 depletion, viability was considerably reduced in cells that had undergone EMT (Figure S6E), suggesting that cancer cells that have undergone EMT are more dependent on LARP6 for supporting their protein synthesis. These results are complementary with recent findings, which have shown a link between EMT and enhanced rRNA transcription (Prakash et al., 2019), and highlight a potential therapeutic avenue, via LARP6 inhibition, for specific targeting of ribosome biogenesis in cancers with strong EMT features.

## Discussion

**Figure 7:**
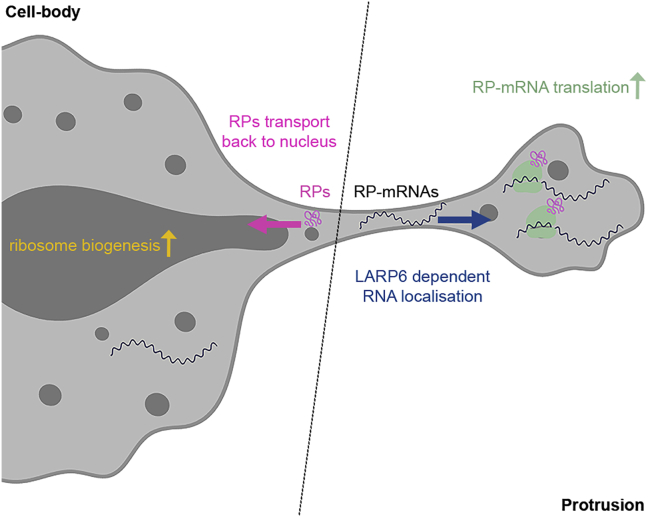
Proposed Mechanism of Ribosome Biogenesis Regulation by LARP6-Dependent RP-mRNA Localization For a Figure360 author presentation of this figure, see https://doi.org/10.1016/j.devcel.2020.10.006. LARP6 binds RP-mRNAs and localizes them to the protrusive fronts of migrating mesenchymal-like cells, where their translation is enhanced due to the local enrichment of active translation machinery. Once translated, nascent RPs transport back to the nucleus to participate in ribosome biogenesis, leading to increased ribosome production and augmented overall protein synthesis.

It is now clear that rather than being uniformly distributed throughout the cytoplasm, the majority of eukaryotic mRNAs exhibit specific subcellular localizations (Benoit Bouvrette et al., 2018; Lécuyer et al., 2007; Wang et al., 2012; Wilk et al., 2016). Such localization can act as a means of localizing the encoded proteins (Zappulo et al., 2017), or function instead as a mechanism for post-transcriptional regulation of gene expression by modulating the access of mRNAs to different trans-acting factors (Kejiou and Palazzo, 2017). Here, we reveal a mechanistic link, based on mRNA localization, between mesenchymal-like cell migration and regulation of ribosome biogenesis. We demonstrate that as cells protrude into their surrounding matrix, RP-mRNAs become enriched at the leading fronts via LARP6, where they come into contact with the locally enriched translation machinery. This compartmentalization results in upregulation of RP-mRNA translation, with the newly synthesized RPs then traveling back to the nucleus to participate in ribosome biogenesis. Ultimately, LARP6-dependent RP-mRNA localization results in upregulation of ribosome biogenesis, leading to enhancement of overall protein synthesis (Figure 7). We propose that this enhancement acts as a feedforward mechanism, enabling mesenchymal-like cells to then produce the large quantities of required proteins to support sustained movement and proliferation. Local synthesis of RPs may also assist their correct folding, as the protein folding machinery is also enriched in protrusions (Mardakheh et al., 2015). In fact, as many RPs are highly charged and contain significant unstructured portions, ribosome biogenesis is known to be particularly reliant on the folding machinery (Karbstein, 2010).

Crucially, a recent study in mammalian gut epithelial cells also demonstrated that the subcellular localization of RP-mRNAs correlated with their translational output, although the molecular mechanism of this localization was not defined (Moor et al., 2017). Instead of the front-back polarity observed in mesenchymal-like migratory cells, gut epithelial cells exhibit apical-basal polarity with distinct protein and mRNA compositions associated with each side of the polarized cell. RP-mRNAs were shown to be primarily localized to the basal portion of the cells in fasting mice but translocated to the apical portion upon feeding where the translation machinery was also enriched, thus, leading to enhancement of their translation in an analogous feedforward mechanism (Moor et al., 2017). It remains to be determined whether LARP6 or another LARP family member is similarly involved in regulation of RP-mRNAs localization in gut cells. Nevertheless, these studies collectively reveal that post-transcriptional regulation by spatial compartmentalization is a previously unappreciated mechanism in controlling RP-mRNA translation and ribosome biogenesis.

In addition to protrusions of mesenchymal-like cells, RP-mRNAs have been found to be highly enriched in axons of different neurons, where they undergo robust translation (Shigeoka et al., 2016). A recent study has shown that certain locally synthesized RPs can get incorporated into pre-existing ribosomes within axons (Shigeoka et al., 2019). While it is possible that such on-site remodeling of ribosomes can also occur in protrusive fronts, our data demonstrate that the bulk of protrusion-synthesized RPs accumulate in the nucleus to participate in canonical ribosome biogenesis. Unlike axons, protrusions are only a few dozen microns away from the nucleus, which makes retrograde transfer of RPs readily achievable. It remains to be shown whether RP-mRNA localization to axons serves additional functions beyond on-site ribosome remodeling. Moreover, whether LARP6 or another LARP family member is similarly involved in localization of RP-mRNAs to axons remains to be determined.

Hyperactive ribosome biogenesis is a common hallmark as well as a driver of many high-grade cancers (Pelletier et al., 2018; Ruggero and Pandolfi, 2003). Enhanced protein synthesis is particularly important for supporting invasion and metastasis (Hsieh et al., 2012; Mendillo et al., 2012), and it is now evident that various anti-cancer chemotherapies function at least in part by disrupting ribosome biogenesis (Pelletier et al., 2018). Consequently, there has been a surge of interest in identifying more specific ways to target ribosome biogenesis in hope of achieving high anti-tumor activity combined with low genotoxic side effects (Drygin et al., 2011). We here show that in breast carcinomas, LARP6 expression is strongly upregulated by EMT, and cells that have undergone EMT are more dependent on LARP6, suggesting that LARP6 inhibition could potentially be used as a therapeutic strategy to specifically inhibit ribosome biogenesis in EMT associated carcinomas. In addition to being more invasive, such carcinomas often exhibit a greater resistance to standard chemotherapies, collectively resulting in poorer outcome (Dongre and Weinberg, 2019). Importantly, we have shown that a small-molecule compound that interferes with LARP6 RNA-binding activity (Stefanovic et al., 2019) can also inhibit RP-mRNA localization to protrusions. Although the safety, efficacy, and pharmacological properties of this specific compound may not be satisfactory for therapeutic use, our results demonstrate the plausibility of therapeutic targeting of LARP6 by small-molecule inhibitors in the context of inhibiting ribosome biogenesis in mesenchymal/EMT associated cancer subtypes.

## STAR★Methods

### Key Resources Table

**Table:** 

REAGENT or RESOURCE	SOURCE	IDENTIFIER
**Antibodies**		

Rabbit polyclonal anti LARP6	Atlas Antibodies	Cat#HPA049029; RRID: AB_2680604
Mouse monoclonal anti vimentin	Abcam	Cat#ab8978; RRID: AB_306907
Rabbit polyclonal anti GFP	Abcam	Cat#ab290; RRID: AB_303395
GFP-Trap®	ChromoTek	Cat#gtma-20; RRID: AB_2631358
Mouse monoclonal anti E-cadherin	Cell signalling	Cat#3195; RRID: AB_2291471
Mouse monoclonal anti TCF8/ZEB	Cell signalling	Cat#3396; RRID: AB_1904164
Phospho-p70 S6 Kinase (Thr389)	Cell signalling	Cat#9206; RRID: AB_2285392
p70 S6 Kinase	Cell signalling	Cat#2708; RRID: AB_390722
α-Tubulin (DM1A)	Cell signalling	Cat#3873; RRID: AB_1904178
α-Tubulin (11H10)	Cell signalling	Cat#2125; RRID: AB_2619646
α-Tubulin Monoclonal Antibody	Thermo Fisher	Cat#A11126; RRID: AB_221538
GAPDH	Novus Biologicals	Cat#NB300-221;RRID: AB_10077627
Alexa Fluor™ 488 Phalloidin	Thermo Fisher Scientific	Cat#A12379
Rabbit IgG HRP linked	GE Healthcare	Cat#NA934; RRID: AB_772206
Mouse IgG HRP linked	GE Healthcare	Cat#NA931; RRID: AB_772210

**Bacterial and Virus Strains**		

DH5α for cloning	Thermo Fisher Scientific	Cat#18265017

**Chemicals, Peptides, and Recombinant Proteins**		

NuPAGE LDS Sample Buffer	Thermo Fisher Scientific	Cat#NP0008
Pierce ECL Plus Western Blotting Substrate	Thermo Fisher Scientific	Cat#N32132
Click-iT™ Plus OPP Alexa Fluor™ 488 Protein Synthesis Assay	Thermo Fisher Scientific	Cat#C10456
Horse Serum	Invitrogen	Cat#16050-122
Human EGF	Prepotech	Cat#AF-100-15-1000
Hydrocortisone	Sigma-Aldrich	Cat#H0888; CAS: 50-23-7
Cholera Toxin from *Vibrio cholerae*	Sigma-Aldrich	Cat#C8052; CAS: 9012-63-9
Insulin from bovine pancreas	Sigma-Aldrich	Cat#I1882; CAS: 11070-73-8
Crystal violet	Sigma-Aldrich	Cat#C6158; CAS: 548-62-9
DTT	VWR Chemicals	Cat#M109; CAS: 3483-12-3
Iodoacetamide	VWR Chemicals	Cat#786-228; CAS: 144-48-9
Blasticidin S HCl	Life Technologies	Cat#R21001
TGF-β1 human	Sigma-Aldrich	Cat#H8541
Emetine	Sigma-Aldrich	Cat#E2375; CAS: 7083-71-8
Harringtonine	Sigma-Aldrich	Cat#SML1091; CAS: 26833-87-4
Puromycin	Sigma-Aldrich	Cat#P9620; CAS: 58-58-2
ANTI-RNase (15-30 U/μL)	Life Technologies Ltd Invitrogen Division	Cat#AM2692
RNase A, DNase and protease-free (10 mg/mL)	Life Technologies Ltd	Cat#EN0531
AZD8055	Selleckchem	S1555; CAS: 1009298-09-2
Torin	Selleckchem	S2827; CAS: 1009298-09-2
Everolimus	Selleckchem	S1120; CAS: 159351-69-6
Hoechst 33342	Thermo Fisher Scientific	Cat#H3570
HCS NuclearMask™ Blue Stain	Thermo Fisher Scientific	Cat#H10325
C9 (Stefanovic et al., 2019)	ChemBridge	DIVERSet-CL chemical library
SignalStain® Antibody Diluent	Cell Signaling Technologies	Cat#8112S
SignalStain® DAB Substrate Kit	Cell Signaling Technologies	Cat#8059P
SignalStain® Boost IHC Detection Reagent (HRP, Rabbit)	Cell Signaling Technologies	Cat#8114P
Antigen Unmasking Solution, Citric Acid Based	Vector Laboratories	Cat#H-3300
DPX new	Merck	Cat#100579
RNAscope® Fluorescent Multiplex Reagent Kit	Advanced Cell Diagnostics Srl	Cat#320850
Hs-RPL34 targeting 24-702 of NM_000995.4	Advanced Cell Diagnostics Srl	Cat#504031
Hs-RPS7 targeting 2-521 of NM_001011.3	Advanced Cell Diagnostics Srl	Cat#504211
Hs-RPLP2	Advanced Cell Diagnostics Srl	Cat#511391
Hs-RPL22	Advanced Cell Diagnostics Srl	Cat#435271
Hs-RPS21	Advanced Cell Diagnostics Srl	Cat#511381
Hs-ITGB4	Advanced Cell Diagnostics Srl	Cat#300031
Edit-R CRISPRa crRNA Non-targeting control, 5nmol	Dharmacon	Cat#U-009500-01-05
Edit-R CRISPR-Cas9 Synthetic tracrRNA, 5 nmol	Dharmacon	Cat#U-002005-05
Edit-R Modified Synthetic crRNA, desalted/deprotected, 2 nmol: LARP6; chr15:70832948(+) - custom design	Dharmacon	Cat#crRNA-409669
DharmaFECT Duo	Dharmacon	Cat#T-2010-01
CellTracker™ Green CMFDA Dye	Thermo Fisher Scientific	Cat#C7025
CellTracker™ Orange CMTMR Dye	Thermo Fisher Scientific	Cat#C2927
Collagen I	Advanced BioMatrix	Cat#5005
Lipofectamine 2000 Transfection Reagent-1.5 mL	Life Technologies	Cat#11668019
Lipofectamine™ RNAiMAX Transfection Reagent	Thermo Fisher Scientific	Cat#13778150
Opti-MEM I Reduced Serum Medium-100 mL	Thermo Fisher Scientific	Cat#31985062
Gibco™ DMEM w/High Glucose and w/o Glutamine, Lysine and Arginine	Fisher Scientific	Cat#12817552

**Critical Commercial Assays**		

TMTsixplex™ Isobaric Label Reagent Set	Thermo Fisher Scientific	Cat#90061
TMT10plex™ Isobaric Label Reagent Set	Thermo Fisher Scientific	Cat#90110
Subcellular Protein Fractionation Kit	Thermo Fisher Scientific	Cat#78840
Pierce High pH Reversed-Phase Peptide Fractionation Kit	Life Technologies	Cat#84868
QuantSeq mRNA 3’ end sequencing kit	Lexogen	Cat#SKU: 015.24
CellTiter-Glo® Luminescent Cell Viability Assay	Promega	Cat#G7571
RNeasy Mini Kit	QIAGEN	Cat#74104
MTT Cell Viability Assay	Thermo Fisher Scientific	Cat#M6494
Qubit™ RNA HS Assay Kit	Thermo Fisher Scientific	Cat#Q32852
Pierce™ BCA Protein Assay Kit	Thermo Fisher Scientific	Cat#23225
High Sens. RNA ScreenTape Sample Buffer	Agilent Technologies	Cat#5067-5580
High Sensitivity RNA ScreenTape	Agilent Technologies	Cat#5067-5579
Brilliant II SYBR® Green QRT-PCR	Agilent Technologies	Cat#600825
MycoAlert™ PLUS Mycoplasma Detection Kit	Lonza	Cat#LT07-705

**Deposited Data**		

TMT quantitative proteomics analysis of (a) protrusions and cells bodies of MDA-MB231 cells collected after 1, 2, 4, & 8 hrs post protrusion induction, and (b) protrusions and cells bodies of a panel of five normal and malignant human cell-lines.	This paper	PXD021239 accessible via PRIDE partner repository (http://www.ebi.ac.uk/pride/archive/)
TMT quantitative proteomics analysis of MDA-MB231 cells grown on either closed or open pore 3 μm transwells for either 2 or 24 hrs.	This paper	PXD021206 accessible via PRIDE partner repository (http://www.ebi.ac.uk/pride/archive/)
Pulsed SILAC quantification of translation rates between MDA-MB231 cells grown on either closed or open pore 3 μm transwells for 1, 2, 4 or 8 hrs.	This paper	PXD021203 accessible via PRIDE partner repository (http://www.ebi.ac.uk/pride/archive/)
Pulsed SILAC coupled with iBAQ quantification of protein abundances within different subcellular locations in MDA-MB231 cells, following protrusion induction.	This paper	PXD021205 accessible via PRIDE partner repository (http://www.ebi.ac.uk/pride/archive/)
TMT quantitative proteomics analysis of non-targeted control siRNA (NT) or LARP6 siRNA transfected MDA-MB231 cells, grown overnight on either closed (no protrusions) or open pore (with protrusions) 3 μm transwells.	This paper	PXD021180 accessible via PRIDE partner repository (http://www.ebi.ac.uk/pride/archive/)
SILAC proteomics analysis of non-targeted (NT) vs LARP6 siRNA treated MDA-MB231 cells.	This paper	PXD021204 accessible via PRIDE partner repository (http://www.ebi.ac.uk/pride/archive/)
3’ mRNA-seq (QUANTSEQ FWD) sequencing of protrusion and cell body fractions of BJ, PC-3M, RPE-1, U-87 and WM-266.4 cells.	This paper	E-MTAB-8470 accessible via ArrayExpress repository (http://www.ebi.ac.uk/arrayexpress)
3’ mRNA-seq (QUANTSEQ FWD) sequencing of protrusion and cell body fractions of non-transfected control siRNA or LARP6 siRNA transfected MDA-MB231 cells.	This paper	E-MTAB-9520 accessible via ArrayExpress repository (http://www.ebi.ac.uk/arrayexpress)
GFP-LARP6 and GFP control sequencing of iCLIP libraries generated from stably expressing MDA-MB231 cells.	This paper	E-MTAB-9636 Accessible via ArrayExpress repository (http://www.ebi.ac.uk/arrayexpress)

**Experimental Models: Cell Lines**		

MDA-MB231	ATCC	ATCC number 92020424
RPE-1	N/A	Dr Sarah McClelland (Barts Cancer Institute)
BJ	N/A	Dr Sarah McClelland (Barts Cancer Institute)
U-87	N/A	Dr Paul Huang (Institute of Cancer Research)
WM-266.4	N/A	Prof. Chris Marshall (Institute of Cancer Research)
PC-3M	N/A	Dr Prabhakar Rajan (Barts Cancer Institute)
MCF10AT	N/A	Dr Susana Godinho (Barts Cancer Institute)

**Oligonucleotides**		

SIRNA UNIV NEGATIVE CONTROL	Sigma-Aldrich	Cat#SIC001
ON-TARGETplus Non-targeting Pool	Dharmacon	Cat#D-001810-10-05
LARP6 siRNA-1 GGAUUCAUGGCCAUGAGA	Sigma MISSION	Cat#Hs02_00351818
LARP6 siRNA-2 GCAAGAUGCUCCUGGUCUA	Sigma MISSION	Cat#Hs01_00153597
LARP6 siRNA-3 CUGUGUAUAAAUACCUUCU	Sigma MISSION	Cat#Hs01_00153598
LARP1 siRNA CUGACUAUGAGAUUGAUGA	Sigma MISSION	Cat#Hs01_00168468
LARP1B siRNA GAGAAUGAUACACGAAGU; AGACCUGGAUCCCGGAACA; UCAAGUAAUCAACGUAAGA; GGUGGUAAUAUCCGAGGUU	Dharmacon	SMARTpool: ON-TARGETplus L-013350-02-0005
LARP7 siRNA AGGAAACAGUCCGGGAUA; GUGCUAUCAAAGAGCGAAU; GCAAAGACUCAACAAGCGA; CUUAAUCAGCCUCGGGAAA	Dharmacon	SMARTpool: ON-TARGETplus L-020996-01-0005
SSB siRNA GGUCGUAGAUUUAAAGGAA; GGUUAGAAGAUAAAGGUCA; GAGACCAGUAGUUUAGUAA; GGGAAGUACUAGAAGGAGA	Dharmacon	SMARTpool: ON-TARGETplus L-006877-01-0005
h47S rRNA fw 5’ TTCGTTCGCTCGCTCGTT 3’	Sigma	N/A
h47S rRNA rv 5’ CAACGACACGCCCTTCTTTC 3’	Sigma	N/A
Hs_LARP6_1_SG QuantiTect Primer Assay	QIAGEN	Cat#QT00221445
Hs_GAPDH_1_SG QuantiTect Primer Assay	QIAGEN	Cat#QT00079247

**Recombinant DNA**		

TetO-WT-L32TOP-β-Globin-12xMS2 (WT 5’TOP reporter)	Gift from A. Gentilella	N/A
TetO-MUT-L32TOP-β-Globin-12xMS2 (MUT 5’TOP reporter)	Gift from A. Gentilella	N/A
MCP-EGFP expression plasmid	Gift from C. Gallego	N/A
VSV lentiviral packaging vectors	Gift from C. Gallego	N/A
deltaR lentiviral packaging vectors	Gift from C. Gallego	N/A
rtTA-N144 plasmid (Richner et al., 2015)	Gift from A. Yoo	Cat#66810; RRID: Addgene_66810
pTK-Twist lentiviral inducible expression plasmid (Guo et al., 2012)	Gift from B. Weinberg	Cat#36977; RRID: Addgene_3697
pTK-Snail lentiviral inducible expression plasmids (Guo et al., 2012)	Gift from B. Weinberg	Cat#36976; RRID: Addgene_3697
pcDNA™6.2/N-EmGFP-DEST Vector	Thermo Fisher Scientific	Cat#V35620

**Software and Algorithms**		

MaxQuant	N/A	https://www.biochem.mpg.de/5111795/maxquant
Perseus	N/A	https://www.biochem.mpg.de/5111810/perseus
BlueBee	N/A	https://www.bluebee.com/
Galaxy	N/A	https://usegalaxy.org/
GradPad PRISM v7	N/A	https://www.graphpad.com/scientific-software/prism/
iMAPS webserver	N/A	https://imaps.genialis.com/iclip
ImageJ	N/A	https://imagej.net/

**Other**		

75 mm Transwell with 3.0 μm pore polycarbonate membrane insert	Corning	Cat#3420
24 mm Transwell with 3.0 μm pore polycarbonate membrane insert	Corning	Cat#3414
6.5 mm Transwell with 3.0 μm pore polycarbonate membrane insert	Corning	Cat#3415
Vivacon 500, 30,000 MWCO Hydrosart	Sartorius	Cat#VN01H22
iBiDi μ-Slide 18 Well flat, ibiTreat	Thistle Scientific	Cat#81826
iBiDi μ-Plate 96 Well Black	Thistle Scientific	Cat#89626
Falcon™ Chambered Cell Culture Slides	Thermo Fisher Scientific	Cat#354118

### Resource Availability

#### Lead Contact

Further information and requests for resources and reagents should be directed to and will be fulfilled by the Lead Contact, Faraz Mardakheh (f.mardakheh@qmul.ac.uk).

#### Materials Availability

Cell lines generated in this study could be made available upon request to lead contact.

#### Data and Code Availability

The mass spectrometry raw files and their associated MaxQuant output files generated during this study are available at ProteomeXchange Consortium (Vizcaíno et al., 2014) via the PRIDE partner repository (http://www.ebi.ac.uk/pride/archive/), as listed in the Key Resources Table. In addition, all RNA-sequencing FASTQ files generated during this study are available at ArrayExpress database (http://www.ebi.ac.uk/arrayexpress), as listed in the Key Resources Table. The accesssion numbers for the mass spectrometry datasets reported in this paper are PRIDE: PXD021203, PXD021204, PXD021205, PXD021206, PXD021239, and PXD021180. The accesssion numbers for the RNA-sequencing datasets reported in this paper are ArrayExpress: E-MTAB-8470, E-MTAB-9520, and E-MTAB-9636.

### Experimental Model and Subject Details

#### Cell Culture

MDA-MB231, U87, and WM266.4 cells (all of female origin) were grown in DMEM supplemented with 10% FBS, 1% Penicillin/Streptomycin. RPE cells (female origin) were grown in DMEMF12, supplemented with 10% heat inactivated FBS, 1% Penicillin/Streptomycin; HEK293T and BJ cells (female and male origin, respectively) were grown in DMEM supplemented with 10% heat activated FBS, 1% Penicillin/Streptomycin; PC-3M cells (male origin) were grown in RPMI supplemented with 10% heat inactivated FBS, 1% Penicillin/Streptomycin; MCF10AT cells (female origin) were grown in DMEMF12 supplemented with 5% horse FBS, 1% Penicillin/Streptomycin, 100 ng/ml cholera toxin, 20 ng/ml epidermal growth factor, 10 mg/ml insulin and 0.5 mg/ml hydrocortisone. All cells were grown in humidified incubator at 37°C with 5% CO_2_, and routinely passaged twice per week. All cell-lines were authenticated by STR profiling (Public Health England) and were routinely checked to be mycoplasma-free by MycoAlert Plus mycoplasma detection kit (Lonza). Cell lines are listed in Key Resources Table.

### Method Details

#### Reagents and Plasmids

The TetO-WT-L32TOP-β-Globin-12xMS2 (WT 5’TOP reporter) and TetO-MUT-L32TOP-β-Globin-12xMS2 (MUT 5’TOP reporter) constructs were a gift from Antonio Gentilella (IDIBELL, Barcelona). MCP-EGFP expression plasmid, as well as the VSV and deltaR lentiviral packaging vectors were a gift from Carme Gallego (IBMB, Barcelona). rtTA-N144 (Richner et al., 2015) was a gift from Andrew Yoo (Addgene plasmid # 66810). pTK-Twist & pTK-Snail lentiviral inducible expression plasmids (Guo et al., 2012) were a gift from Bob Weinberg (Addgene plasmids #36977 & #36976). GFP-LARP6 expression plasmid was generated by Gateway cloning of a custom synthesized codon-optimized human LARP6 donor vector (GeneArt) into the pcDNA6.2_N-EmGFP-DEST vector (Thermo). LARP6 expression constructs were verified by DNA sequencing. Edit-R Cas9 expression plasmid with puromycin resistance was purchased from Dharmacon. The C9 compound was acquired as part of a compound library from ChemBridge. Reagents used in this study are listed in Key Resources Table.

#### 3D Collagen-I RNA-FISH

Collagen-I gel matrix was prepared as described previously (Mardakheh et al., 2015), with slight modifications. Briefly, 5x DMEM adjusted with 0.1M NaOH and 3.7% NaHCO_3_ was mixed with pepsinized bovine collagen-I (Advanced BioMatrix) and diluted with dH_2_O to 1.7 mg/ml of Collagen-I whilst on ice. The mixture was then poured into individual wells of iBidi μ-Slide with 18 wells and allowed to set at 37°C for 2 hrs. Subsequently, the cells were plated on the top of the set matrix in complete media. After 2 days of cell invasion through the collagen-I gels, cultures were fixed with 10% Neutral Buffered Formalin (NBF) for 30 min before further processing for dual RNA-FISH and antibody staining, and imaging by confocal microscopy.

#### 3D Collagen-I Invasion Assay

3D Collagen-I invasion assays were performed as described previously (Mardakheh et al., 2015), with some modifications. Briefly, cells were suspended in 2.3 mg/ml serum-free pepsinized bovine collagen-I (Advanced BioMatrix) to a final concentration of 100,000 cells/ml. For each condition, 200μl of cell suspension was dispensed into a well of an iBidi 96-μ-plate 96 Well Blackwell plate, pre-coated with 0.2% fatty acid free BSA. 4 wells were used per condition as technical replicates. Plates were then centrifuged at 300 g to collect the cells at the bottom, before incubating the plate at 37°C/10% CO_2_ for 2 hrs to allow the Collagen to set over the cells. Subsequently, 60 μl of DMEM/10% FBS was added to the top of each well to trigger invasion of the cells upward. Cells were allowed to invade overnight at 37°C/10% CO_2_, before being fixed and stained with addition of 8% formaldehyde in PBS, supplemented with 5 μg/ml Hoechst (Thermo). The plates were then imaged on a Nikon spinning disk confocal microscope with 20X magnification, using 5x5 tile scans at 0 μm, 20 μm, 40 μm, 60 μm, 80 μm, 100 μm, and 120 μm z-planes relative to the bottom of each well.

#### siRNA Transfections

For siRNA-mediated depletions, 10,000 cells/cm^2^ were seeded on standard TC-treated polystyrene plates overnight. Transfections were conducted using Lipofectamine RNAiMAX and Opti-MEM (Thermo), according to manufacturer’s instructions, at a final concentration of 20 nM siRNA. Cells were analyzed 72 hrs post transfection, or as indicated if otherwise. siRNA sequences used in this study are listed in Key Resources Table.

#### Lentivirus Production and Transduction

Lentiviral particles were produced in HEK293T cells by co-transfection of indicated lentiviral vectors plus packaging VSV and deltaR vectors. 1,000,000 HEK293T cells were seeded in one well of a 6 well plate 6 hrs prior to the transfection. The transfection was performed using Lipofectamine 2000 (Thermo) with 2 μg of the lentivirus vector and 1 μg of each of the packaging vector, according to manufacturer’s instructions. The transfection mix was then added to the medium of the cells for 12-14 hrs, before removal and addition of 3 ml of fresh DMEM supplemented with 30% FCS, L-Glu, P/S, for virus production. After 24 hrs, the lentivirus containing medium was harvested and passed through a 0.45um filter. Half of the supernatant was then used to reverse transduce 50,000 MDA-MB231 cells in a 6 well plate.

#### MS2 Reporter Generation and Imaging

The MS2 reporter was generated by engineering MDA-MB231 cells to express rtTA, MCP-GFP, and the WT or MUT 5’TOP reporter constructs, via Lentiviral transduction and DNA transfection, combined with antibiotic selection, single clone selection, and FACS sorting. Briefly, rtTA-N144 lentiviral particles were produced in HEK293T cells as described above and used to reverse transduce 50,000 MDA-MB231 cells. 72 hrs post transduction, the medium was exchanged with fresh DMEM containing 500 μg/ml Hygromycin B for antibiotic selection. The selection was continued whilst keeping the confluency of the cells below 50% and refreshing the selection medium every 3 days for ∼2 weeks until all the cells in the negative control well were dead. Single colonies were generated using the surviving population of MDA-MB231 cells by diluting 50 cells in 10 ml medium and dispersing 100 μl in each well of a 96 well plate. Verified rtTA-N144 expressing MDA-MB231 clones were then transfected with 2.5 μg of the MCP-GFP vector in a six well plate, using Lipofectamine 2000 (Thermo) according to manufacturer’s instructions. Two days after transfection, the cells were selected with 1,500 μg/ml of G418 for ∼2 week until all the cells in the negative control well were dead. 5,000,000 of the G418 selected cells were then FACS sorted to enrich for a cell population with high GFP signal, followed by generation of single colonies as mentioned above. Finally, WT or MUT 5’TOP vectors were integrated into the stable rtTA-N144 and MCP-GFP expressing MDA-MB231 clones through Lipofectamine 2000 transfection as before, using 2.5 μg of the vectors. 2 days post transfection, the cells were treated with 0.5 μg/ml of Puromycin for ∼10 days until all the cells in the negative control well were dead. As described before, single colonies were generated from the surviving population. A successful incorporation of the 5’TOP constructs was later verified through qPCR analysis of β-Globin expression induction following doxycycline treatment. Live-cell imaging was carried out on a Nikon spinning disk confocal microscope with 100X magnification.

#### CRISPR Knockout Generation

LARP6 CRISPR/Cas9 knockout cells were generated according to Dharmacon’s Edit-R CRISPR-Cas9 Gene Knockout platform, using a custom-made crRNA sequence against Exon 3 of human LARP6 (5’ACAAGTAGAGATCATAGACC3’), along with trcRNA, and a Cas9 expression plasmid with puromycin resistance, all acquired from Dharmacon. The Cas9 plasmid, trcRNA, and LARP6 or a non-targeting control crRNA, were co-transfected into MDA-MB231 cells using DharmaFECT Duo (Dharmacon), according to manufacturer’s instructions. After 48-72 hrs, the cells were selected for 48 hrs with 0.5 μg/ml puromycin. Single colonies were generated using the surviving population of MDA-MB231 cells by diluting 50 cells in 10 ml medium and dispersing 100 μl in each well of a 96 well plate. Successful knockout single clones were then identified by western blotting with LARP6 antibody. A total of 3 non-targeting and 15 LARP6 knockout clones were screened by western blotting, and two non-targeting and two LARP6 KO clones were selected for downstream experiments.

#### Generation of Stable GFP-Expressing Cells

MDA-MB-231 cells were transfected with expression constructs containing GFP or GFP-LARP6, using Lipofectamine2000 (Thermo) according to manufacturer’s instructions, and selected with 10 μg/ml blasticidin for 7 days prior to FACS sorting to enrich for medium to high level GFP expressing cells.

#### Protrusion Purification

Cell protrusions were fractionated as described before (Mardakheh et al., 2015), with some modifications. 10 million cells were seeded on top of 5 μg/ml collagen-I coated 75 mm polycarbonate transwell filters with 3-μm pore size (Corning), and allowed to adhere overnight without the addition of media to the bottom chamber of the transwells. The next day, the media on the top of the filter was replaced by fresh media, and protrusions were induced by addition of the same media to the bottom chamber for indicated times. For RNA-sequencing, transwells were then washed with RNase- free PBS, and RNA was purified from protrusions by shaving the bottom of the filter using a glass coverslip dipped in RLT buffer from RNeasy Mini Kit (QIAGEN). The Cell-body fraction was subsequently collected by direct addition of the RLT buffer to the top of the filter. RNA was extracted following manufacturer’s instructions and quantified by Qbit RNA HS Assay Kit (Thermo). For proteomics analysis, multiple transwells were washed by PBS, fixed with methanol for 20 min at -20°C, washed again with PBS, and the protrusions were shaved off using a glass coverslip dipped in lysis buffer (2% SDS, 100 mM Tris/HCl pH 7.5). Cell body fractions were prepared by direct addition of the lysis buffer to the top of the filter. Protein amounts were estimated by Pierce BCA Protein Assay Kit (Thermo) prior to sample preparation for MS.

#### RNA-FISH, Immunofluorescence (IF), and Confocal Microscopy

For staining of 3D invading cells, 300,000 cells grown for 2 days on 3D Collagen-I matrix filled wells of iBidi u-Slides were used. For staining of cells on 2D, 5,000 cells grown on Collagen-I coated Falcon multi-chamber slides were used. For staining of cells that protrude through transwell, 1,000,000 or 100,000 cells seeded onto 24 mm or 6.5 mm membrane inserts respectively, were used. For RNA-FISH, cells were washed with RNase-free PBS and fixed in RNase-free NBF for 30 min. The fixed cells were then washed three times with RNase-free PBS and dehydrated gradually with 50%, 70% and 100% ethanol. Cells were subsequently rehydrated gradually with 70% and 50% ethanol in RNase-free PBS, and treated with RNAScope® Protease III for 10 min prior to hybridization with pre-designed RNAScope® probes (Advanced Cell Diagnostics). All probes were then visualized using RNAscope® Fluorescent Multiplex Reagent Kit according to the manufacturer’s protocol (Advanced Cell Diagnostics). If co-immunofluorescence was also conducted, samples were blocked after RNA-FISH with 10% BSA in RNase-free PBS for 20 min, and incubated with the indicated antibodies overnight at 4°C, followed by incubation with secondary antibody for 1hr at room temperature (RT). The images were acquired on Zeiss LSM 710 or 880 confocal microscopes. Imaging of protrusion and cell-body sides of transwell filters was done as described before (Mardakheh et al., 2015), with the filters being visualized by transmitted light imaging in grey. 3x3 tiled confocal scans were acquired as large field of view images. All used antibodies in this study are listed in Key Resources Table.

#### RiboPuromycylation-FISH Assay

Ribopuromycylation assay was performed as described in (Bastide et al., 2018), with some modifications. Briefly, transwells or slides were treated with labelling medium containing 25 μg/ml emetine plus puromycin 50 μg/ml for 5 min at 37°C. The medium was then aspirated and slides were incubated for 20 min with ice-cold co-extraction/fixation buffer (0.015% digitonin, 5 mM MgCl_2_, 25 mM KCl, 0.2 M sucrose, 1x EDTA-free protease inhibitors, 1/1000 ANTI-RNase, 3% Formaldehyde, and 50 mM Tris-HCl pH 7.5, in RNase-free water). The slides were then further fixed in 10% NBF for 10 min at RT. The fixed cells were then washed three times with RNase-free PBS, followed by RNA-FISH and IF staining with RPL34 RNAScope® probe and anti-puromycin antibody, and imaging by confocal microscopy.

#### Immunohistochemistry (IHC)

A cohort of 33 Formalin-Fixed Paraffin-Embedded (FFPE) human breast carcinoma specimens consisting of 26 Invasive Ductal Carcinoma (IDC) and 7 Metaplastic breast carcinoma (MBC) samples, retrieved from the Barts Cancer Institute Breast Tissue Bank following full informed consent (ethics ref: 15/EE/0192) were analyzed by IHC. Standard 3,3′-Diaminobenzidine (DAB) method for immunostaining combined with low-pH citrate based high-pressure cooking antigen retrieval was used as reported in (Mao et al., 2010), with some modifications. Briefly, tissues were sectioned and affixed onto coated slides before being subjected to deparaffinization (two washes in xylene for 5 min) and rehydration (two washes in absolute alcohol for 2 min). Endogenous peroxidase was blocked by immersing the tissues in methanol 0.03% hydrogen peroxide in methanol twice for 5 min. Two additional washes in absolute alcohol was performed to clear out any remaining regents and sections were then rinsed under tap water. Subsequently, sections were heated in antigen unmasking solution (Vector labs) in a pressure cooker, reaching boiling point for 10 min and then cooled for 5 min under tap water. Sections were then dried and a hydrophobic pen was used to draw marks around tissues before transfer to wash buffer (0.2% tween in PBS). Subsequently, sections were incubated in blocking solution (2.5% bovine serum albumin and 0.2% tween in PBS) for 1 h. Next, LARP6 primary antibody (Atlas antibodies, product number: HPA049029, lot number: R58965) diluted in SignalStain® Antibody Diluent (Cell Signaling Technologies) was added and incubated overnight at 4°C in a wet chamber. Next day, SignalStain® Boost Detection Reagent was equilibrated to RT. Antibody solution was removed and the sections were washed with wash buffer for 3 times. Sections were then incubated with SignalStain® Boost Detection HRP rabbit reagent (Cell Signaling Technologies) in a humidified chamber for 30 min at RT, before being washed again for three times and incubation with SignalStain® DAB for 10 min, followed by immersion in water for 5 min and counterstaining with haematoxylin for 2 min. Stained sections were then dehydrated in 90% absolute alcohol for 2 min and transferred to xylene for 5 min for clearing, before mounting of a cover glass using DPX mounting medium. Specimens were then dried and visualized using an OLYMPUS BX51 microscope.

#### OPP Staining

OPP staining and detection was conducted using Click-iT Plus OPP Alexa Fluor-488 Protein Synthesis Assay Kit (Thermo), according to manufacturer’s instructions. Briefly, cells were treated with 10 μM OPP for 15 min at 37°C, before being fixed with 4% formaldehyde for 15 min at RT, washed three times with PBS, and permeabilized for 5 min with 0.2% Triton X-100 in PBS. The cells were then washed three times with PBS, and the OPP labelled nascent proteins were detected using Click-iT® mediated covalent attachment of Alexa Fluor-488 azide dye. Cell were then counterstained with phalloidin (to detect cell boundaries) and NuclearMask blue (Thermo) during a 30 min incubation at RT, before three further PBS washes and imaging by confocal microscopy.

#### Image Analysis

Immunofluorescence images were analyzed using ImageJ or Fiji software platforms (Schindelin et al., 2012). For quantification of RNA-FISH in transwells, multi-channel color images were split, intensity levels were thresholded, followed by normalization RNA-FISH signal to the overall cell-body or protrusion areas. Protrusion and cell-body areas were defined by either CellTracker staining (Thermo), tubulin IF staining, or phalloidin labelling. Normalized protrusion to cell-body RNA-FISH values were then calculated and displayed in Log_2_ scale. For presentation of images, cell boundaries were marked by white dash-lines generated in the Zen blue software (Zeiss). Polarity index (Park et al., 2012) was used as a quantification of RNA localization to the cell peripheries, and was calculated as PI= $\frac{\sqrt{(\left(\bar{x}\text{RNA}-\bar{x}\text{cell}\right)2+(\bar{x}\text{RNA}-\bar{x}\text{cell})2}}{\text{Rgcell}}$, where x̅RNA and $\bar{x}$RNA are the transcript pixel intensity positions and $\bar{x}$cell and $\bar{y}$cell are the positions for the nucleus centroid. Rgcell is the radius of gyration and it is calculated by the root-mean-square distance of all transcript pixels from the nucleus centroid. Co-localization analyses were performed by ComDet plugin. ComDet plugin was also used to detect and quantify the number of MCP-GFP labelled 5’TOP reporter mRNA particles from every frame image of protrusion videos. For quantification of translation, mean OPP fluorescence intensity of the cell-body images were normalized to their corresponding DAPI image intensity. LARP6 IHC staining of tumor sections were quantified using the IHC Profiler ImageJ plugin (Varghese et al., 2014). This plugin allows for the color deconvolution of haematoxylin (blue) and DAB (brown) pixels. Briefly, “Nuclear Stained Image” mode was selected to find nuclei and threshold was manually set to ensure selection of malignant cells. H DAB channel overlapping with malignant cells was selected for analysis on IHC Profiler using the “Cytoplasmic Stained Image” mode. IHC Profiler macro outputs of ‘high positive’ and ‘positive’ were collectively grouped as ‘strongly positive’, whilst the ‘low positive’ output was referred to as ‘weakly positive’. The over-representation in metaplastic carcinomas was calculated using Fisher’s exact test, with a *P*-value cut-off of 0.05.

#### Western Blotting

Cell were lysed in 2-4% SDS, 100mM Tris/HCl pH 7.5 and sonicated with a sonicator bath (Bioruptor Pico - Rm 343) for 15 cycles. Sample concentration was adjusted with a Pierce BCA Protein Assay Kit (Thermo) before addition of NuPAGE LDS Sample Buffer (Thermo) with reducing agent and boiling at 95°C for 10 minutes. After separation on a NuPAGE 4%–12% Bis/Tris protein gel (Thermo), proteins were transferred to an Immobilon-P membrane (Millipore) using a standard wet transfer device. Primary antibodies were diluted in 5% BSA, PBS and incubated on the membranes at 4°C overnight followed by incubation with anti-mouse or rabbit HRP-conjugated secondary antibodies at room temperature for one hour. Membranes were then probed with Pierce ECL Plus HRP-detection reagent followed by imaging on an Amersham Imager 600. All used antibodies in this study are listed in Key Resources Table.

#### Colony Formation and Cell Viability Assays

For Colony formation assay of non-targeting control or LARP6 siRNAs transfected cells, 72 hrs post transfection, 5,000 cells were seeded in 6-well TC-treated plates and allowed to grow for 10 days. Cells were then fixed with 4% formaldehyde for 30 min at 4°C in the dark. The fixing solution was then discarded and a 0.5% crystal violet solution (0.5% w/v; 20% MeOH; 80% ddH_2_O) was added to the plates and incubated for 10 min at RT, before extensive washing of the plates with water. Colony images were taken with an Amersham Imager 600 machine (GE Healthcare Life Sciences). The Crystal Violet stain was then extracted with Sorenson’s buffer (0.1M Na_3_C_6_H_5_O_7_; 50% EtOH; 50% ddH_2_O), left on agitation at 300 rpm for 30 min. Colorimetric quantification was conducted by measuring absorbance at 540 nm with a FLUOstar Omega Microplate Reader (BMG Labtech). Each biological replicate was measured in 3 technical replicates. At least 3 biological replicates were performed to calculate the average OD value. For assessment of cell viability with CellTiter-Glo® (Promega) luminescence assay, 5,000 cells/cm^2^ were transfected with non-targeting control or indicated LARP6 siRNAs. Three, five, or seven days post-transfection, CellTiter-Glo™ reagent was added (150 μl of per well of 24 well plates). The plates were then shaken for 2 min, incubated for 10 minutes, and RLU were measured with a FLUOstar Omega Microplate Reader (BMG Labtech). Each biological replicate was measured in 3 technical replicates. At least 3 biological independent replicates were performed to calculate the average RLU value. For assessment of cell viability after C9 treatment, WT and LARP6 KO cells were seeded 24 hrs prior experiment into 96 well TC-treated plates and consequently treated with C9 for 48 hrs at indicated concentrations. IC50 measurements were calculated using MTT assay (Thermo) according to manufacturer’s instructions. Readouts were normalized and IC50 values were calculated using a non-linear regression model. Each biological replicate was performed in 4 technical replicates. At least 3 biological independent replicates were performed to calculate the average IC50 value.

#### RT-qPCR

RT-qPCR was performed using Brilliant II SYBR® Green one-step (Agilent) with the ABI 7500 Real-Time PCR system (Applied Biosystems). The 2-ΔΔCT method was used for relative quantification of genes expression according to (Rao et al., 2013). GAPDH was used as internal control for normalization. LARP6 expression reduction on KDs was validated by RT-qPCR. All primers for RT-qPCR are listed in Key Resources Table.

#### Transcriptomics Analysis

RNA was extracted using RNeasy kit (QIAGEN), and total RNA preparations were quantified by a Qubit 4 fluorimeter (Thermo). Quality of RNA was analyzed on Agilent Tapestation 4200 with High Sens. RNA ScreenTape to rule out RNA degradation (RIN ≥8). Libraries were prepared from 50-100 ng of RNA using Lexogen QuantSeq FWD mRNA 3’ end sequencing kit (Lexogen), according to manufacturer’s instruction. Libraries were sequenced on an Illumina Nextseq 500, at Barts and the London Genome Centre. FASTQ files from QuantSeq 3’ mRNA-seq data were aligned to the human reference genome using BlueBee Genomics platform. Raw read count data were uploaded into Perseus software (Tyanova et al., 2016b) for downstream data analysis, including log_2_ scaling, protrusion to cell-body ratio calculation, normalization by median subtraction, Benjamini-Hochberg corrected 1D or 2D annotation enrichment analysis (Cox and Mann, 2012), and data visualization. Galaxy platform (Afgan et al., 2018) was used to validate knockdown of isoform specific reads of LARP6 which were not differentiated by the BlueBee platform analysis.

#### iCLIP

The iCLIP method was performed as previously described in (Huppertz et al., 2014), with the following conditions. A total of ∼ 40 million cells per biological replicate of GFP and GFP-LARP6 stably expressing MDA-MB-231 cells were irradiated once on ice with 150 mJ/cm^2^ of UVC (254 nm), using a Hoefer Scientific UV Crosslinker. A total of 4 replicates of GFP and 6 replicates of GFP-LARP6 were irradiated. Cell pellets were lysed in iCLIP lysis buffer and diluted to a protein concentration of 1mg/ml. RNA is fragmented in lysate with RNase I at 0.4 U/ml. GFP or GFP-LARP6 was immunoprecipitated with GFP (ab290) or GFP-trap magnetic agarose beads (Chromotek). After SDS-PAGE and membrane transfer, the region corresponding to 75–200 kDa protein-RNA crosslinked complexes was excised to isolate the associated RNAs. Isolated RNAs were reverse transcribed using primers containing an experimental barcode (5nt, underlined) and UMI sequence: /5Phos/ WWW XXXXX NNNN AGATCGGAAGAGCGTCGTGAT /iSp18/ GGATCC /iSp18/ TACTGAACCGC. Samples were sequenced on Illumina HiSeq4000, producing 100-nt single-end reads. For data analysis, individual GFP and GFP-LARP6 iCLIP FASTQ files were uploaded onto the iMaps webserver (https://imaps.genialis.com/), which is based on the iCount package (https://icount.readthedocs.io/ en/latest/index.html), for demultiplexing and primary analysis. Reads were mapped to the GRCh38/GENCODE v27 genome. Crosslink sites were defined as the nucleotide position preceding the start of the cDNA insert (i.e. where the reverse transcription truncates). Sequencing reads arising from PCR duplication were removed by collapsing reads which map to the same crosslink site position and contain the same UMI sequence. Analysis of reproducibility of crosslink sites between biological replicates was performed by PCA, implemented in R using gene counts values. iCount group function was used to merge 6 replicates of GFP-LARP6 and 4 replicates of GFP individual BED files, coming from two independent biological experiments, into one BED file per condition. Reads density bar-plots were generated using the iCount summary type and subtype outputs. Metaprofile of crosslink counts normalized to total library size of the merged GFP and GFP-LARP6 replicates were plotted as RNA maps around gene start, gene end, and ORF start landmarks. Peak calling was performed using the Paraclu (Frith et al., 2008) function within iMaps, with the minimal sum of scores inside a cluster set to 10, maximal cluster size set to 200 nucleotides, and Minimal density increase set to 2. GFP peaks were subtracted from GFP-LARP6 peaks using the bedtools intersect function in Galaxy (Afgan et al., 2018) to reveal LARP6 specific binding sites. LARP6 specific target mRNAs were identified on the basis of at least having one specific LARP6 binding site. Fisher’s exact test analysis of over-represented categories amongst LARP6 specific targets were performed in Perseus software (Tyanova et al., 2016b), using an FDR cut-off of 0.02.

#### Stable Isotope Labelling of Amino Acids in Cell Culture (SILAC)

For SILAC labelling, cells were grown for at least six doublings in Lysine and Arginine free DMEM, supplemented with 10% dialyzed FBS, 1% P/S, 600mg/L Proline, in the presence of 100mg/L of either light Arginine and Lysine (for ‘‘light’’ media), medium Arginine [U-13C6] and Lysine [4,4,5,5-D4] (for ‘‘medium’’ media), or heavy Arginine [U-13C6, U-15N4] and Lysine [U-13C6, U-15N2] (for ‘‘heavy” media). For pulsed SILAC, cells were grown in light SILAC media overnight, before being switched to fresh medium or heavy SILAC media for 1 to 8 hrs. After lysis, sonication, and protein concentration assessment, equal amounts of SILAC or pulsed SILAC samples were reciprocally mixed. For pulsed SILAC in conjugation with subcellular fractionation, cells were pulsed for 4 hrs with either heavy or medium labels, before lysis and mixing, followed by subcellular fractionation with serial solubilization.

#### Mass Spectrometry Sample Preparation, Data Acquisition, and Analysis

Lysates, prepared in 2-4% SDS, 100mM Tris/HCl pH 7.5, were reduced with addition of 100 mM DTT and boiling at 95°C for 10 min. Filter Aided Sample Preparation (FASP) (Wiśniewski et al., 2009) was used for generation of tryptic peptides in case of label-free or SILAC/pulsed SILAC samples. For TMT samples, isobraric Filter Aided Sample Preparation (iFASP) (McDowell et al., 2013) was performed, with some modifications. Briefly, 25 μg of total protein for each sample was reduced with 50 mM Bond-Breaker TCEP Solution (Thermo) at 95°C for 10 min. Reduced samples were then diluted in UA buffer (8 M urea, 100 mM Tris HCl pH 8.5), and transferred to Vivacon 500 Hydrosart filters with a molecular cut-off of 30kDa, before being concentrated by centrifugation at 14,000 g for 20 min. Samples were then washed twice with urea (UA) buffer through cycles of buffer addition and concentration, before alkylation with addition of 10 mM iodoacetamide in UA buffer at RT for 30 min in the dark. Samples were then washed three additional times with the UA buffer, before two washes with 100 mM TEAB to reduce the urea concentration. Samples were then trypsin digested overnight at 37°C in a 600 rpm shaking thermomixer, using 100 μL of 100mM TEAB supplemented with 0.5 μg Trypsin (Sigma) per filter. Each Sample was then supplemented with 0.2 mg of a TMT label reagent at 25°C for 1 hr, followed by quenching with 5% hydroxylamine at 25°C for 30 min. Peptides were eluted by centrifugation at 14,000 g for three times, plus a further elution with 30% acetonitrile. After combining all eluates, the samples were dried with a vacuum concentrator and fractionated using Pierce™ High pH reverse-phase fractionation kit into 7 fractions, according to manufacturer’s instructions. Samples were then dried with vacuum centrifugation before LC-MS/MS analysis. LC-MS/MS analysis was performed on a Q Exactive-plus Orbitrap mass spectrometer coupled with a nanoflow ultimate 3000 RSL nano HPLC platform (Thermo Fisher). Dried peptide mixtures were resuspended in 0.1% TFA, 2% Acetonitrile, and ∼1-5 μg of total material was injected into the nanoflow HPLC. Samples were resolved at flow rate of 250 nL/min on an Easy-Spray 50cm X 75 μm RSLC C18 column (Thermo Fisher). Each run consisted of a 123 min gradient of 3% to 35% of Buffer B (0.1% FA in Acetonitrile) against Buffer A (0.1% FA in LC-MS gradient water), and separated samples were infused into the MS by electrospray ionization (ESI). Spray voltage was set at 1.95 kV, and capillary temperature was set to 255°C. MS was operated in data dependent positive mode, with 1 MS scan followed by 15 MS2 scans (top 15 method). Full scan survey spectra (m/z 375-1,500) were acquired with a 70,000 resolution for MS scans and 17,500 for the MS2 scans. For TMT10plex samples, MS2 scans were acquired with 35,000 resolution. A 30 sec dynamic exclusion for fragmented peaks was enabled.

MaxQuant (versions 1.5.5.1 and 1.6.3.3) was used for all mass spectrometry search and quantifications (Tyanova et al., 2016a). Raw data files were searched against a FASTA file of the Homo sapiens proteome, extracted from Uniprot (2016). Enzyme specificity was set to “Trypsin”, allowing up to two missed cleavages. False discovery rates (FDR) were calculated using a reverse database search approach, and was set at 1%. Default MaxQuant parameters were used with some adjustments: For TMT experiments, “reporter ion MS2” type option was selected with a reporter mass tolerance of 0.01 Da. TMT 6plex or 10plex isobaric labels were selected according to the experiments. For SILAC experiments, “Match between runs” option was enabled. With the exception of pulsed SILAC experiments, the “Re-quantify” option was also enabled. A minimum ratio count of 1 was also used for pulsed SILAC experiments. The iBAQ calculation was also selected for nuclear, cytosol, and membrane abundance calculation of newly synthesized RPs. All downstream data analyses, such as data filtering, Log 2 transformation, ratio calculation, category annotation, 1D & 2D annotation enrichment analysis, and data visualization, were performed in Perseus software (Tyanova et al., 2016b) (versions 1.5.5.3 and 1.6.2.1). For all annotation enrichments, GO and KEGG annotations were used, with a Benjamini-Hochberg FDR of < 0.02 applied as the cut-off in the adapted Wilcoxon Mann-Whitney test.

### Quantification and Statistical Analysis

Details of statistical analysis and the number of replicates can be found in the figure and dataset legends.

## Supplemental Information

Document S1. Figures S1–S6

Document S2. Datasets S1–S16

Figure360. An Author Presentation of Figure 7

Document S2. Article plus Supplemental Information
