# Supplementary material for: Subcellular mRNA Localization Regulates Ribosome Biogenesis in Migrating Cells
Source: Dev Cell. 2020 Nov 9;55(3):298–313.e10. doi: 10.1016/j.devcel.2020.10.006 (PMC7660134; doi:10.1016/j.devcel.2020.10.006)
Supplement: Document S1. Figures S1–S6 [file mmc1.pdf]

Developmental Cell, Volume *55*

Supplemental Information

Subcellular mRNA Localization Regulates

Ribosome Biogenesis in Migrating Cells

Maria Dermit, Martin Dodel, Flora C.Y. Lee, Muhammad S. Azman, Hagen Schwenzer, J. Louise Jones, Sarah P. Blagden, Jernej Ule, and Faraz K. Mardakheh

A

Cell-line	RP-mRNAs enrichment score (maximum = 1)	RP-mRNAs enrichment FDR (Benjamini- Hochberg)	RP-mRNAs mean ratio (Log2)
MDA-MB231	0.889867	8.13E-40	1.78997
U87	0.364976	6.04E-08	0.498162
PC3-M	0.296722	1.05E-05	0.315796
WM266.4	0.567795	3.56E-17	0.790841
RPE1	0.627752	1.18E-20	0.756156
BJ	0.426182	2.53E-10	0.537236

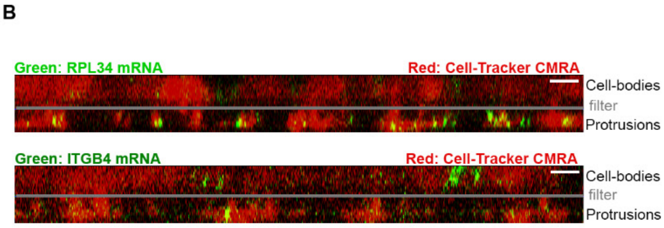

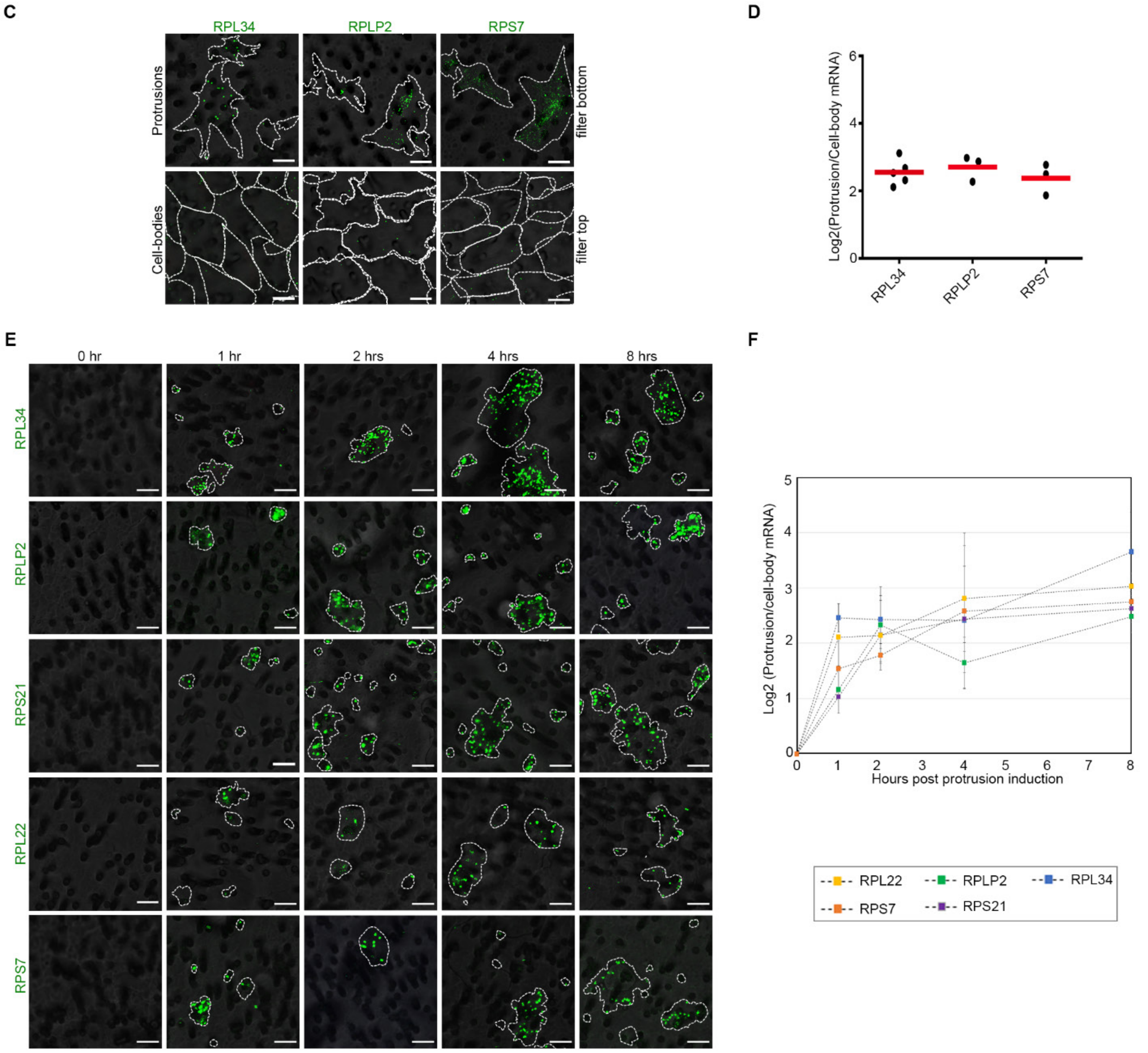

Figure S1, related to Figure 1: RP-mRNAs universally localize to protrusions. (A) RP-mRNAs are significantly enriched in protrusions of every tested cell-line. Results of 1-dimensional annotation enrichment analysis (Cox and Mann, 2012) of data presented in Figure 1C (Dataset S1), showing the average Log2 of protrusion to cell-body ratio values for RP-mRNAs, the Benjamini-Hochberg FDR significance of their protrusion enrichment, and their calculated enrichment score, in each indicated cell-line. **(B)** Protrusions formed through transwell filters are enriched in RPL34 mRNA whereas cell-bodies are enriched in ITGB4 mRNA. Cross-section views of confocal Z- stack images of MDA-MB231 cells protruding through 3-µm transwell filters. Cells were stained with CellTracker (red), fixed and analyzed with RNA-FISH for RPL34 mRNA or ITG4B mRNA (green). Grey lines mark the position of the polycarbonate filter. **(C)** RNA-FISH validation of protrusion localization of indicated RP-mRNAs in RPE1 cells.

Representative RNA-FISH images of protrusions and cell-bodies of RPE1 cells, stained with probes against the indicated mRNAs (Green). Cell boundaries (dashed lines) were defined by co-staining of the cells with anti-tubulin antibody. The filters (grey) were visualized by transmitted light microscopy. **(D)** Quantification of protrusion to cell-body RNA-FISH ratio values from experiments shown in (C), showing protrusion enrichment. Ratio values from a total of 3-5 large field of view images were quantified per each probe. **(E)** RP-mRNA localization to protrusions occurs as early as 1 h post-induction of protrusions and persists over time. Representative FISH images of five RP-mRNAs (green) in protrusions (dash- lines) of MDA-MB231 cells at indicated times. **(F)** Quantification of RNA enrichments in protrusions from experiments shown in (E). A total of 3 large fields of view per time-point per probe were imaged and quantified. All scale bars are 10 µm.

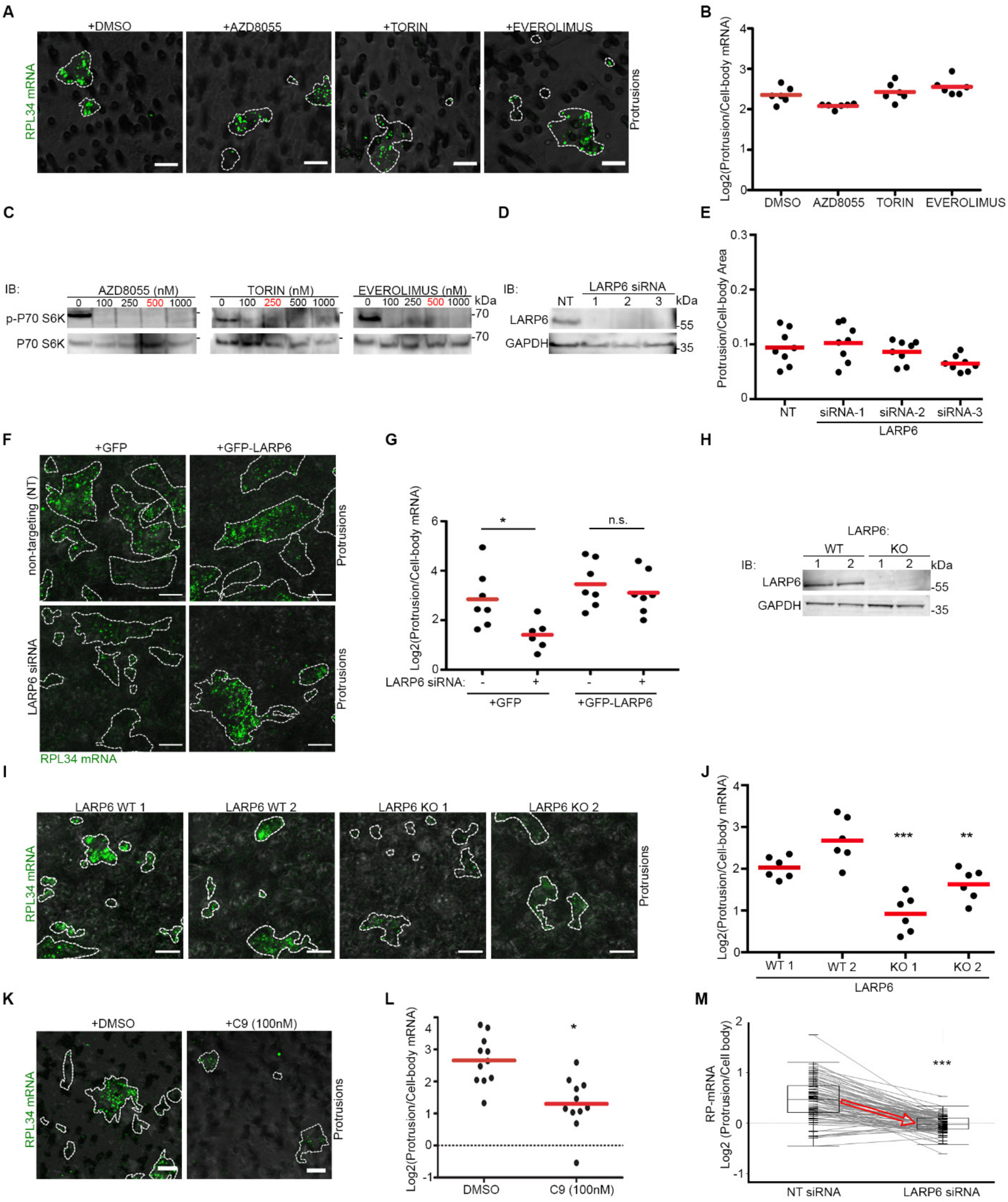

Figure S2, related to Figure 2: LARP6 localizes RP-mRNAs to protrusions. (A) Localization of RP-mRNAs is independent of mTORC1. Representative RNA-FISH images of RPL34 mRNA (Green) in protrusions (dash-lines) of non-treated vs. mTORC1 inhibitor treated MDA-MB321 cells for 2 hrs. The transwell filters (grey) were visualized by transmitted light microscopy. **(B)** Quantification of RPL34 mRNA in protrusions of experiments shown in (A). A total of 6 large fields of view per condition from two independent experiments were imaged and quantified. **(C)** Validation of mTORC1 inhibitors by immunoblotting (IB). MDA-MB231 cells were treated for 2 hrs with indicated doses of mTORC1 inhibitors and assessed by IB for the phosphorylation status of P70 S6 Kinase. Inhibitor concentrations highlighted in red were used for the localization analyses in Figure S2A. p-P70 S6K: Phospho-p70 S6 Kinase (Thr389). **(D)** Validation

of siRNA mediated LARP6 KD by immunoblotting (IB). MDA-MB231 cells were transfected with the indicated siRNAs for 72 h before IB with the indicated antibodies. **(E)** LARP6 depletion does not impair the ability of cells to form protrusions, allowing quantification of mRNA localizations between control and LARP6 knockdown cells in a comparable manner. Relative protrusion/cell-body areas in MDA-MB231 cells transfected with non-targeting (NT) control siRNA or three independent LARP6 siRNAs from experiments shown in Figure 2D. **(F)** Expression of an siRNA resistant LARP6 construct rescues RP-mRNA localization to protrusions. Representative images of RPL34 mRNA (green) in protrusions of MDA-MB231 cells stably expressing GFP or GFP-LARP6, transfected with NT control siRNA or an siRNA against 3'UTR of endogenous LARP6 (i.e. siRNA-3). Protrusion boundaries (dash-lines) were defined by co-staining with anti-tubulin antibody. The transwell filters (grey) were visualized by transmitted light microscopy. **(G)** Quantification of protrusion to cell-body ratio values for RPL34 mRNA from experiments shown in (F). 7 large field of view images per condition from two independent experiments were quantified. n.s.: non-significant; *$P<0.05$. **(H)** Validation of LARP6 CRISPR/Cas9 knockout (KO) by IB. Two independent clones of LARP6 WT and KO MDA-MB231 cells were lysed and analyzed by IB with indicated antibodies. **(I)** LARP6 KO cells fails to enrich RPL34 mRNAs in protrusions. Representative RNA-FISH images of RPL34 mRNA (Green) distribution in protrusions (dash-lines) of indicated MDA-MB321 cells. The transwell filters (grey) were visualized by transmitted light microscopy. **(J)** Quantification of RPL34 mRNA enrichment in protrusions from experiments shown in (I). A total of 6 field of view images per condition from two independent experiments were quantified. *P*-values were calculated using two-tailed, homoscedastic t-test. **$P<0.01$; ***$P<0.001$. **(K)** Treatment of transwell protruding cells with LARP6 inhibitor (C9) blocks RP-mRNA localization to protrusions. Representative RNA-FISH images of RPL34 mRNA in protrusions of MDA-MB231 that were allowed to protrude through transwells for 2 hrs in presence of 100 nM C9 or DMSO. Protrusion boundaries (dash-lines) were defined by co-staining with anti-tubulin antibody. **(L)** Quantification of RPL34 mRNA enrichment in protrusions from experiments shown in (K). 11 large field of view images per condition were quantified. *$P<0.05$. All scale bars are 10 µm. **(M)** Quantification of protrusion to cell-body ratio values of RP-mRNAs from experiments shown in Figure 2H-J. Box plot of RP-mRNA ratio values from NT control and LARP6 siRNA treated MDA-MB231 cells, showing loss of protrusion enrichment upon LARP6 depletion. Error bars are min-max range. Significance p-values were calculated using a two way t-test analysis. ***$P<0.001$.

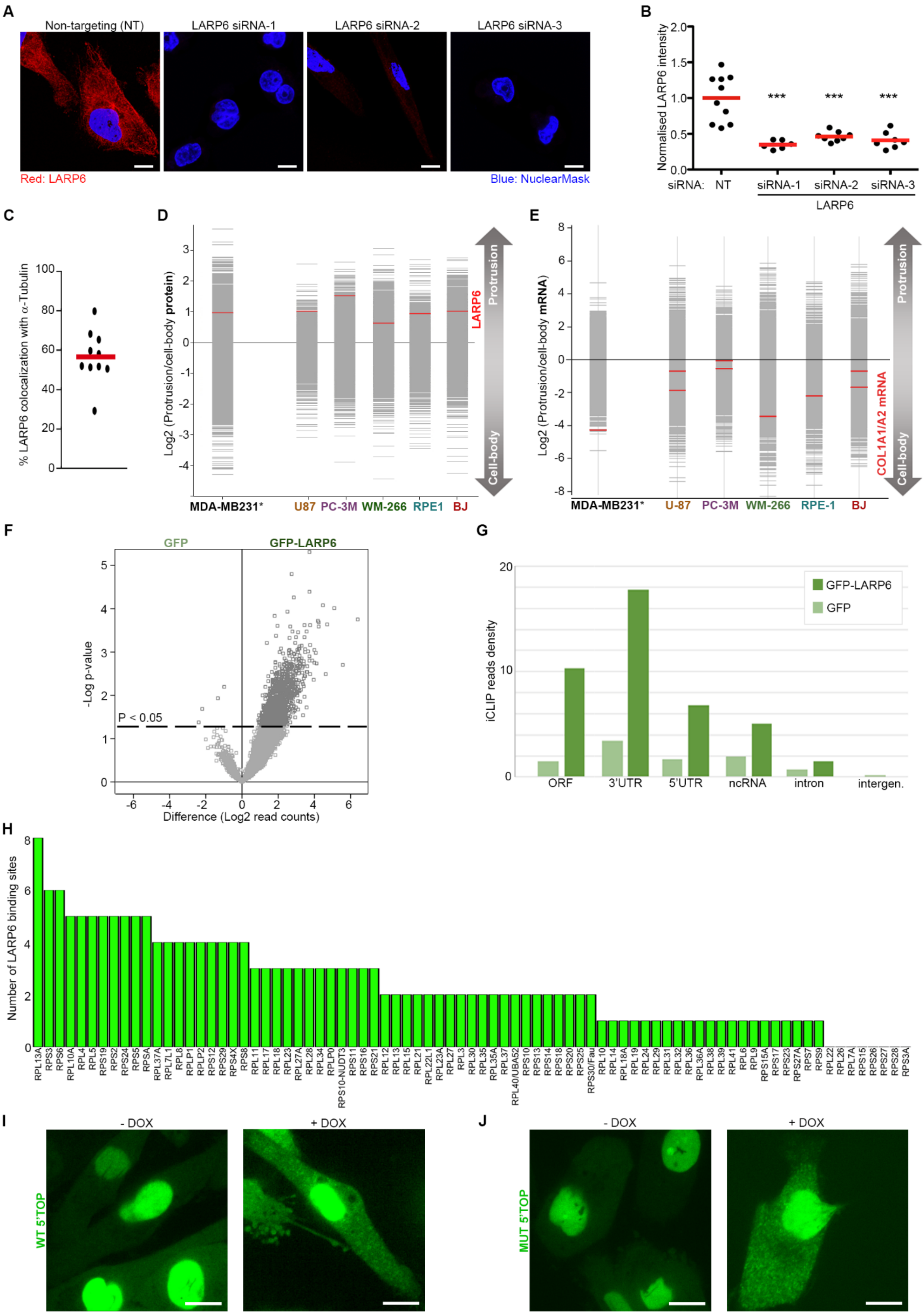

Figure S3, related to Figure 3: LARP6 is a protrusion-enriched RBP which directly binds RP-mRNAs. (A) Validation of LARP6 antibody by IF. Representative IF images of LARP6 IF staining in MDA-MB231 cells transfected with NT control or indicated siRNAs for 72 h. **(B)** Quantification of

relative LARP6 fluorescence intensity from experiments shown in (A). A total of 6-10 large field of views were quantified. P-values were calculated using two-tailed, homoscedastic t-test. ****P*<0.01. **(C)** LARP6 co-localizes with microtubules. Percentage of LARP6 signal co-localizing with α-Tubulin, in IF experiments shown in Figure 3A. A total of 10 field of view images from two independent experiments were quantified. **(D)** LARP6 protein is enriched in protrusions of all the cell-lines investigated. Log2 of protrusion/cell-body protein levels, measured by TMT proteomics (Dataset S2), were plotted as line column plots, with LARP6 highlighted in red. *MDA-MB231 data was obtained from (Mardakheh et al., 2015). **(E)** Collagen-I mRNAs are mainly enriched in the cell bodies of the cell-lines investigated. Log2 of protrusion/cell-body mRNA levels, measured by RNA-seq (Dataset S1), were plotted a line column plots, with COL1A1 and COL1A2 mRNAs highlighted in red. *MDA- MB231 data was obtained from (Mardakheh et al., 2015). **(F)** Volcano plot comparison of iCLIP transcript read counts between GFP and GFP-LARP6 pulldowns. *P*-values were calculated by a two-sample t-test analysis. Transcripts with significantly higher read counts (*P*<0.05) are marked in dark grey. The vast majority of identified transcripts have significantly higher read counts in the GFP-LARP6 pulldowns. **(G)** iCLIP read densities corresponding to different genomic regions from GFP and GFP-LARP6 iCLIP experiments. **(H)** The majority of RP-mRNAs contain two or more LARP6 binding sites. Bar-graph of the number of called peaks corresponding to specific LARP6 binding sites per each RP-mRNA. **(I)** In WT 5'TOP MS2 reporter containing MDA- MB231 cells, GFP-MCP exhibits a diffuse cytosolic staining with enrichment in the nucleus in the absence of doxycycline (-DOX), but cytoplasmic RNA particles can be observed in the presence of doxycycline (+DOX). **(J)** In MUT 5'TOP MS2 reporter containing MDA- MB231 cells, GFP-MCP exhibits a diffuse cytosolic staining with enrichment in the nucleus in the absence of doxycycline (-DOX), but cytoplasmic RNA particles can be observed in the presence of doxycycline (+DOX). All scale bars are 10 µm.

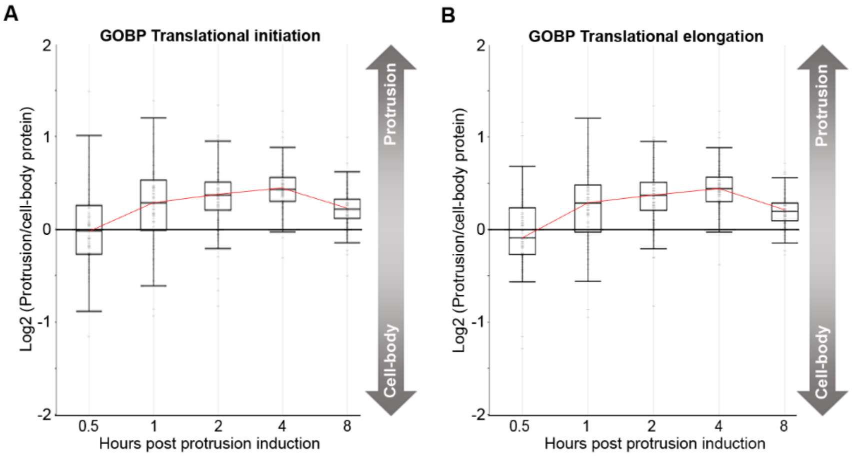

C

Fraction	Enriched GOCC Category	Category Size	Enrichment Score (Maximum = 1)	Enrichment FDR (Benjamini-Hochberg)
Cytosol	cytosol	1560	0.302234	2.72E-60
	cytoplasm	1933	0.204933	3.34E-30
	extracellular membrane-bounded organelle	1304	0.187718	2.56E-21
	extracellular organelle	1304	0.187718	2.42E-21
	extracellular vesicular exosome	1304	0.187718	2.30E-21
Nucleus	nuclear part	2041	0.396308	6.19E-125
	nucleoplasm	1460	0.345676	2.94E-81
	nucleoplasm part	518	0.476506	4.73E-69
	chromosomal part	385	0.43222	1.96E-43
	nucleus	1949	0.234198	3.94E-43
Membrane	integral to membrane	939	0.578113	1.60E-164
	intrinsic to membrane	953	0.574196	1.96E-164
	membrane part	1480	0.419601	4.93E-118
	organelle membrane	1210	0.329221	2.78E-64
	mitochondrial part	544	0.446442	7.95E-63

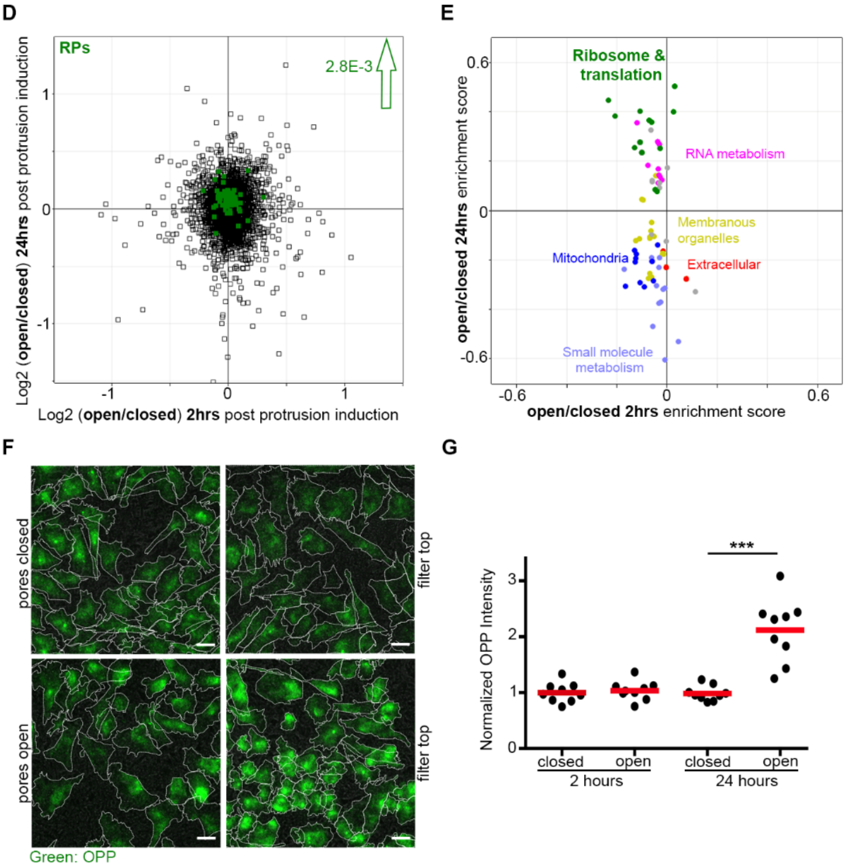

Figure S4, related to Figure 4: Protrusion localization of RP-mRNAs enhances their translation and ribosome biogenesis. (A) Time-course changes in the distribution of proteins annotated in Gene Ontology Biological Pathways (GOBP) database as ‘translational initiation’, between

protrusions and cell-bodies of MDA-MB231 cells. Log2 of protrusion/cell-body protein ratio values of the annotated proteins from indicated time-points were plotted. **(B)** Time-course changes in the distribution of proteins annotated in GOBP database as 'translational elongation', between protrusions and cell-bodies of MDA- MB231 cells. Log2 of protrusion/cell-body protein ratio values of the annotated proteins from indicated time- points were plotted. **(C)** Validation of the subcellular fractionation procedure used in Figure 4I. 1D annotation enrichment analysis for GO Cellular Component (GOCC) protein categories on the iBAQ estimated percentages of proteins (L) in each subcellular fraction (Dataset S11), reveals expected enrichment of cytosolic, nuclear, and membrane residing protein categories. Top 5 most significantly enriched GOCC categories in each fraction are reported. **(D)** Protrusion formation enhances total RP levels. Changes in protein levels between cells with or without protrusions, at 2 or 24 hrs post protrusion induction were quantified by TMT proteomics (Dataset S12). RP levels (green) increase in cells upon induction of protrusions for 24 but not 2 hrs. Benjamini-Hochberg corrected P-value of the increase in RP levels after 24 hrs is reported on the graph. **(E)** 2-dimensional (2D) annotation enrichment analysis of data shown in (D). Each data point represents a protein category inferred from GO and KEGG, and similar categories are highlighted with the same colors (Dataset S13). **(F)** Protrusion formation enhances overall protein synthesis. Transwell seeded MDA-MB231 cells were either prevented from protruding through pores (pores closed), or allowed to form protrusions (pores open), for 2 or 24 hrs, before OPP labelling for 15 mins. OPP was then visualized by Click chemistry mediated conjugation of Alexa Fluor-488. Representative images of the cells from top of the filters at indicated time points are displayed. Cell-boundaries (dash-lines) were defined by anti-tubulin staining. Scale bars are 20 µm. **(G)** Quantification of normalized OPP staining levels from experiments shown in (F). A total of 9 large field of view images per condition were quantified. *P*-values were calculated using two-tailed, homoscedastic t-test. ***$P<0.001$.

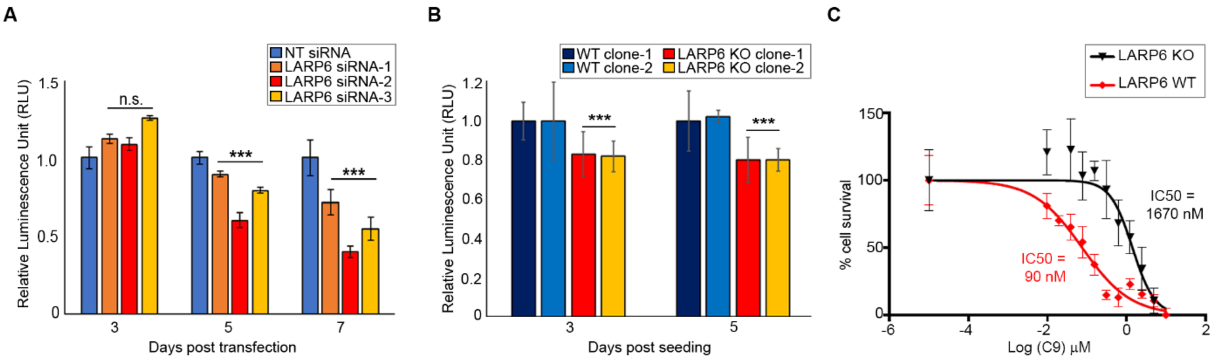

Figure S5, related to Figure 5: Depletion, loss, or inhibition of LARP6 reduces cell viability. (A) Cell viability of MDA-MB231 cells quantified by CellTiter-Glo assay at indicated time-points after transfection with indicated siRNAs. Averages were calculated from 3 biological replicates, each performed in 3 technical replicates. Error bars are SD. *P*-values were calculated using two-tailed, homoscedastic t-test. n.s.: non-significant; ***P<0.001. **(B)** Cell viability quantified by CellTiter-Glo at the indicated time-points post seeding of LARP6 WT or KO cells. Averages were calculated from 3 biological replicates each performed in 3 technical replicates. Error bars are SD. P-values were calculated using two-tailed, homoscedastic t-test. ***P<0.001. **(C)** LARP6 inhibitor (C9) treatment reduces cell-viability in a LARP6 dependent manner. Viability curves for LARP6 WT and LARP6 KO MDA-MB231 cells exposed to a range of concentration of C9 from 0.01 nM to 10 µM for 2 days were determined by MTT assay. Average values were calculated from 3 biological replicates, each performed in 4 technical replicates. IC50 values for each cell-line is reported on the graph. Error bars are SD.

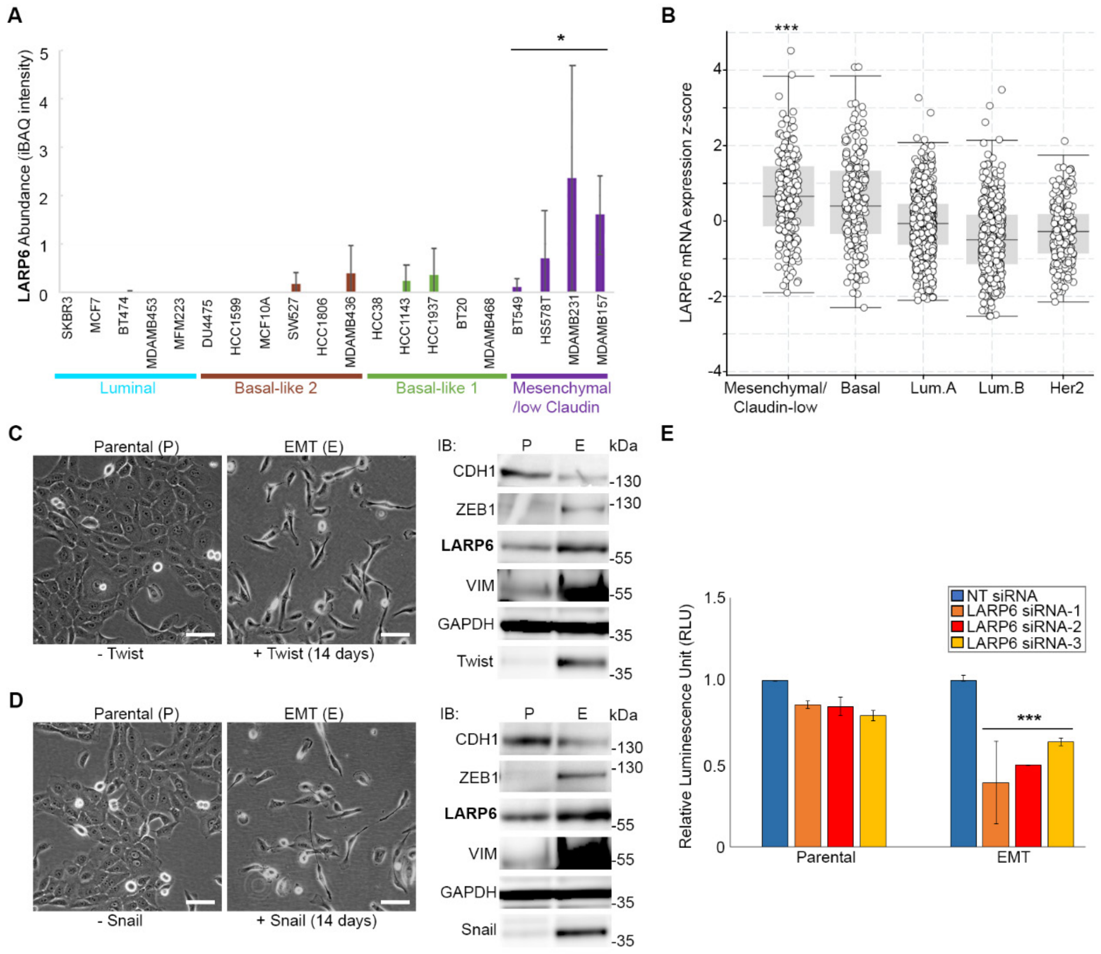

Figure S6, related to Figure 5: LARP6 is triggered by EMT to support cell viability. (A) LARP6 protein is mainly detectable in the Mesenchymal/low Claudin molecular subtype of breast cancer cell-lines. LARP6 absolute protein abundances, calculated by iBAQ, were extracted from (Lawrence et al., 2015) and plotted across the profiled cell-lines belonging to the different molecular subtypes of breast cancer. Error bars are SD. *P*-value was calculated using a one-way ANOVA test. **P*<0.05. **(B)** LARP6 mRNA expression is highest amongst Mesenchymal/low Claudin molecular subtype of breast cancer tumors. LARP6 expression profile of 1758 primary tumors from the METABRIC study (Curtis et al., 2012) was plotted via cBioPortal (Cerami et al., 2012). Classification into intrinsic subtypes was based on their PAM50 clinical attribute. *P*-value was calculated using a one-way ANOVA test. **P*<0.001. **(C)** Induction of EMT by overexpression of Twist upregulates LARP6. LEFT: morphology of MCF10AT cells stably harboring a doxycycline inducible Twist construct, with or without doxycycline treatment (1μg/ml) for 14 days, reveals EMT induction following Twist overexpression. Scale bars are 50 μm. RIGHT: IB analysis of EMT markers (CDH1, ZEB1, VIM), Twist, and LARP6, from the cells shown on the left. GAPDH was used as loading control. **(D)** Induction of EMT by overexpression of Snail upregulates LARP6. LEFT: morphology of MCF10AT cells stably harboring a doxycycline inducible Snail construct, with or without doxycycline treatment (1μg/ml) for 14 days, reveals EMT induction following Snail overexpression. Scale bars are 50 μm. RIGHT: IB analysis of EMT markers (CDH1, ZEB1, VIM), Snail, and LARP6, from the cells shown on the left. GAPDH was used as loading control. **(E)** Cells that have undergone EMT are more sensitive towards LARP6 depletion. Cell-viability of MCF10AT Parental and EMT pairs from Figure 6C, following transfection with indicated siRNAs for 72 hrs, was quantified by CellTiter-Glo assay. Averages were calculated from 3 biological replicates, each measured in 3 technical replicates. Error bars are SD. *P*-values were calculated using two-tailed, homoscedastic t-test. ****P*<0.001.
